# A computational investigation into the role of tRNAs encoded by *Shigella* phage Sf14

**DOI:** 10.1186/s12864-025-11998-9

**Published:** 2025-09-29

**Authors:** Nykki D. Ross, Sarah M. Doore

**Affiliations:** Department of Microbiology and Cell Science, University of Florida, Gainesville, FL 32611 USA

**Keywords:** Bacteriophage, Phage-encoded tRNAs, Codon usage bias, Translational efficiency, Gene expression

## Abstract

**Background:**

Bacteriophage Sf14 infects *Shigella flexneri*, a major foodborne pathogen that causes shigellosis outbreaks primarily in developing nations. It is a Moogle-like myovirus that encodes 26 tRNAs recognizing 19 different amino acids. The presence of tRNAs in phage genomes have been known for decades, but their functions are still poorly understood and appear to vary between species. This work uses computational methods to test several existing hypotheses regarding phage-encoded tRNAs in the context of Sf14 infection. Analyses of codon usage, tRNA adaptation, and tRNA mutation patterns were performed to test four hypotheses of phage tRNA function: codon usage bias, host range expansion, an all-destructive infection phenotype, and escape from host nucleases.

**Results:**

Data from these analyses excluded hypotheses of host range expansion and escape from host anticodon nucleases. Instead, results suggest phage-encoded tRNAs are likely used during late stages of infection, primarily increasing expression of structural genes. While there are significant differences in codon usage bias between Sf14 gene groups and *S. flexneri* 2457T, translational efficiency of Sf14 late genes (as estimated by tRNA adaptation index) is highest when using the phage tRNA pool only.

**Conclusions:**

The most likely hypothesis explaining the presence of tRNAs in the Sf14 genome is the possession of an all-destructive infection phenotype, where genes encoding host tRNAs are degraded along with the genome and translation relies heavily or exclusively on the phage tRNAs. The differences in codon usage also suggest phage-encoded tRNAs specifically affect the production of late gene products.

**Supplementary Information:**

The online version contains supplementary material available at 10.1186/s12864-025-11998-9.

## Introduction

Bacteriophages, also simply called phages, are viruses that infect bacteria. They are highly abundant in every environmental niche in which bacteria are found. Phages greatly outnumber their bacteria hosts in both diversity and population size, with an estimated 10:1 ratio of phage to bacteria [1]. Until recently, most characterized phages of Gram-negative bacteria infected *E. coli* and *Salmonella enterica*. Over the past decade, a number of phages infecting *Shigella flexneri* have been identified and characterized, increasing the depth of understanding of phage biology [2]. *S. flexneri* is a globally problematic pathogen, responsible for 164.7 million cases of bacillary dysentery each year, with 163.2 million of these cases occurring in developing nations [3, 4]. Due to its small inoculum size, rapid transmission, and high rates of antibiotic resistance [4, 5], *Shigella* species are difficult to combat as pathogens. For example, a sustained shigellosis outbreak occurred in Michigan in 2016 throughout eight months in two counties [6].

Around this time, a total of 16 novel *Shigella*-infecting phages were isolated nearby [7]. Of these phages, seven were classified as Moogle-like phages infecting *S. flexneri*, having genetic and structural features similar to Citrobacteriophage Moogle. These phages carry an unusually large number of tRNA genes, typically 25–27, in their 85–95 kbp genomes. Among these isolates was phage Sf14, which encodes 26 tRNAs clustered together in a 4.6 kbp region of the 87.6 kbp genome [2].

The presence of transfer RNAs (tRNAs) in bacteriophage genomes was first described in 1968, when the *Escherichia coli* phage T4 was shown to carry eight genes encoding tRNAs [8–10]. Despite this, little is known about phage-encoded tRNAs and their roles in infection. Four hypotheses have been proposed. First, the codon usage bias (CUB) hypothesis states that phage-encoded tRNAs serve to compensate for codon bias differences between a phage and its host [11–13]. All genomes exhibit a bias in their codon usage, with certain synonymous codons being used preferentially over others [14–16]. Since phages require host components to propagate themselves, it was assumed that the phage would have a similar codon usage bias to the host for optimal efficiency. In phages encoding tRNAs, however, this is not always the case, suggesting that phage-encoded tRNAs compensate for codons used frequently by the phage but infrequently by the host. A second hypothesis that branched off from the CUB hypothesis is that host ranges can expand with the acquisition of tRNAs. The expanded host range hypothesis states that the presence of tRNAs in phage genomes serve to increase a phage’s host range by allowing the phage to infect hosts with varying codon usage [17, 18].

While codon usage compensation and host range expansion are the leading theories behind the presence of phage-encoded tRNAs, hypotheses three and four were recently proposed. These include the all-destructive infection hypothesis proposed by Yang et al. [19]; and the nuclease escape hypothesis proposed by van den Berg et al. [20]. Yang et al. determined that an all-destructive infection phenotype drove tRNA acquisition in the Vibriophage NPV2275O. Since the entirety of the host genome is degraded during infection, including genes encoding tRNAs, NPV2275O must supply its own tRNAs to continue phage replication following depletion of the host tRNA pool. This was further evidenced by the shift in tRNA pool adaptation during infection, with the late-expressed genes favoring the phage-encoded tRNA pool over the host tRNA pool [19]. The nuclease escape hypothesis proposed by van den Berg et al. was based on the discovery of anticodon loop mutations in several mycobacteriophage-encoded tRNAs. During infection, the host bacterium produces anticodon nucleases to target and degrade their own tRNAs [21], which limits the resources available to the phage. The experiments by van den Berg et al. showed these anticodon loop mutations render the phage-encoded tRNAs resistant to degradation, thus allowing infection to proceed despite the degradation of host tRNAs [20].

Computationally, several measurements for analyzing codon usage bias and translational efficiency can be employed [22, 23] to differentiate between these four hypotheses. The relative synonymous codon usage (RSCU) measures which codons are preferred for each amino acid. A high RSCU for a given codon suggests it is used preferentially within a gene or genome, while a low RSCU indicates it is rarely used. Preferred or frequently-used codons are thought to correlate with the relative abundances of isoaccepting tRNAs, resulting in increased translational efficiency of these codons [24]. Another metric, the tRNA adaptation index (tAI), measures the influence of tRNAs on translational efficiency by indicating how well an organism’s tRNA pool correlates with its codon usage [25–27]. The tAI values range from 0 to 1 and are calculated for each gene in an organism, with higher values indicating higher levels of expression and more efficient translation.

It has already been determined that Sf14 can infect multiple serotypes of *S. flexneri* but not other enteric bacteria [7]. Based on this, it is unlikely that the phage tRNAs expand the host range: thus, we excluded the host range expansion hypothesis from further analysis and focused on the remaining three. If the CUB hypothesis can explain the presence of tRNAs in the phage Sf14 genome, one would expect the codon RSCU values to be significantly different between phage and host across their entire genomes and for all genes of interest. Furthermore, we would expect tAI values to be higher than if using only the host tRNA pool for all phage genes. In the case of the all-destructive infection phenotype, we would expect the phage late genes to specifically show high tAI values for its own tRNAs, since the host tRNAs would be absent and therefore not used. Finally, if the phage tRNAs show anticodon loop mutations respective to its host tRNAs, this would suggest phage-encoded tRNAs are escaping host nucleases during infection, keeping them available for phage replication.

To determine which of these three hypotheses can best explain the interactions between Sf14 and its laboratory host *S. flexneri* 2457 T, the RSCU of phage and host genes were calculated and compared. These RSCU values were then used in combination with tRNAscan-SE 2.0 data to calculate the tRNA adaptation of these genes. The tAI when using host, phage, or both tRNA pools was then calculated and compared to simulate the changes in tRNA availability during the all-destructive infection phenotype. Finally, analysis of potential anticodon loop mutations was performed to determine if anticodon nuclease resistance drives tRNA acquisition. While mutations were found in approximately half of the Sf14 tRNAs, they were not suspected to confer resistance to host anticodon nucleases. In addition, *S. flexneri* only encodes the VapC anticodon nuclease, which targets the tRNA for fMet [28], a tRNA not encoded by Sf14, making the biological implications of these mutations unknown. Overall, results suggest Sf14 possesses an all-destructive infection phenotype and primarily relies on its own tRNAs during late stages of infection.

## Materials and methods

### Genomes and identification of genes of interest for phage-host codon usage analysis

The genomes for the host strain *Shigella flexneri* 2457T and the Moogle-like *Shigella* phage Sf14 were downloaded in FASTA format from the National Center for Biotechnology Information (NCBI) GenBank database using accession numbers NC_004741 and NC_042075.

A total of 286 *S. flexneri* 2457 T genes were selected for analysis, each belonging to one of five functional groups – metabolism, biosynthesis, gene expression, stress response and drug resistance, or transport and miscellaneous. Genes were selected by searching previous reports of the 2457 T proteome [29–31]. For Sf14, genes were assigned to one of four groups – early, middle, late, or hypothetical – based on current annotation (v1 10-Jan-2023) or homology with FelixO1 genes [32]. For the three temporal groups, “early” corresponded to host interaction genes and lysis inhibition genes expressed early in infection; “middle” genes were those involved in gene expression, DNA replication and repair; and “late” genes encoded structural or lysis proteins. Despite not having an assigned function, genes encoding hypothetical proteins were included in analysis due to making up a large proportion of the Sf14 genome.

All tRNA genes were identified using the web version of tRNAscan-SE 2.0 (https://lowelab.ucsc.edu/tRNAscan-SE/) [33]. tRNAscan-SE 2.0 data was saved in excel sheets for use in tRNA adaptation index calculations. These data are available in Supplementary Materials.

### Calculation of GC content for phage and host

The total GC content, along with the GC1, GC2, and GC3 content, were calculated in R using the seqinr package ( [34] 2023 update). FASTA files for the genomes and genes of interest were loaded into R for analysis. These values were calculated for the genomes of Sf14 and *S. flexneri* 2457 T, plus each of the various groups of genes listed above. The percentages for total GC, GC1, GC2, and GC3 for both the genome comparison and the group comparison were visualized with bar graphs using the ggplot2 package [35]. The R script used for calculation is available on GitHub (https://github.com/nykkiross/codon-usage).

### Calculation of relative synonymous codon usage for phage and host

Relative synonymous codon usage (RSCU) values for phage Sf14 and its host *S. flexneri* 2457 T were calculated and visualized in R using the following packages: seqinr [34], ggplot2 [35], reshape2 ( [36], 2020 update) pheatmap [37], and factoextra [38]. FASTA files for the genomes of Sf14 and 2457 T were loaded into R for analysis, along with FASTA files for each of the gene groups examined. At the genome level, codon usage was calculated for each individual coding sequence. The RSCU values for each codon were averaged across the genome and visualized using bar plots. For the genes of interest, RSCU was calculated as above and visualized with heatmap and principal component analysis plots. Additionally, the average RSCU for each codon within each gene group was calculated and visualized in R with bar plots. The R scripts used for RSCU calculation and visualization of results are available on GitHub.

### Reformatting of RSCU for tRNA adaptation index analysis

Prior to tRNA adaptation index (tAI) analysis, RSCU data were reformatted to have all codons in uppercase to match the tRNAscan-SE 2.0 data. Reformatting also rearranged the data frame – with the original calculations, each codon had its own column. With the rearrangement, there were five columns – Sequence, Group (or Genome), Codon, RSCU, and Amino Acid. The final format was then appropriate for tAI analysis.

### Calculation of tRNA adaptation index values

Prior to tAI calculation, RSCU values were calculated for *S. flexneri* 2457 T and *Shigella* phage Sf14. Anticodon scores for the tRNAs encoded by each organism were obtained using tRNAscan-SE 2.0 and were loaded into R as anticodon libraries. tRNA scores were normalized to the global maximum tRNA score for each organism, and these normalized scores were used for tAI calculations [25]. Each codon in the RSCU data was converted into its corresponding anticodon using a reverse complementation function. These codons were then matched to the organism-specific anticodon library to calculate the weighted codon value (*S*_*ij*_ weights) for each codon within each organism’s genome [26, 27]. Codon weights were calculated using the following formula:

$$tAI_{weight}\;=\;(\mathrm{RSCU}\;+\;\mathrm\epsilon)\;\mathrm x\;\mathrm{norm}\;\mathrm{tRNA}\;\mathrm{score}$$

where ϵ is a small value (1 × 10^−6^) to prevent division by zero during log calculations [39], RSCU is the relative synonymous codon usage value for a given codon in the genome, and norm tRNA score is the normalized tRNA score for the complementary anticodon. Since the analyzed organisms do not have full anticodon repertoires, any missing anticodons from the anticodon dictionaries were automatically assigned a fallback weight of 0.01.

Next, the geometric mean of all tAI weights was calculated using the following formula:

$$tAI\:=\:exp(\Sigma\log(tAI_{weight})/\mathrm N)$$

where N is the number of matched codons for a given anticodon.

Finally, tAI values were normalized across the genome so that the maximum value is 1:

$${\mathrm{tAI}}_{\mathrm{final}}\;=\;tAI/\max(tAI)$$

where tAI is the calculated tRNA adaptation index value for a given gene and max(tAI) is the maximum tAI across all genes in the genome. For analyzing the tAI of specific gene groups, the tAI for all gene groups were normalized to the tAI values of their respective genomes to avoid artificially inflating values. The average tAI for each organism was calculated by summing all tAI values for an organism and dividing by the number of genes. Data were visualized in R using ggplot2 [35]. The full R script for tAI analysis is available on GitHub.

### Mutation identification and analysis

Potential mutations in Sf14 tRNAs were detected using the readr [40], dplyr [41], and stringr [42] packages in R by comparing the anticodon loop sequences to the sequences in the corresponding host tRNAs. The anticodon and anticodon loop sequences were extracted from each tRNA sequence and compared. Mac Homebrew was used to load the RNAfold package into R [43]. RNAfold was then used to determine secondary structures of host and phage tRNAs, the free energy (∆G) required to destabilize each tRNA, and the difference in ∆G (∆∆G) between each pair of host and phage tRNAs. The ∆G values for each tRNA were then compared to the RSCU values of the corresponding codons for both Sf14 and *S. flexneri* 2457 T and a Pearson’s correlation analysis was run to determine if a relationship between RSCU and tRNA stability existed (data not shown). Analyses were also run to compare the ∆G values of mutated vs. non-mutated tRNAs, as well as consistent vs. inconsistent tRNAs. Models of four representative tRNA mutants were generated using tRNAscan-SE 2.0. The full R script for mutation and free energy analyses is available on GitHub.

### Statistical analyses

All statistical analyses were conducted in R using the packages ggpubr [44], FSA [45], dplyr [41], tidyr [46], and effsize [47]. The package ggplot2 [35] was used to visualize results. To analyze the statistical significance of total GC, GC1, GC2, and GC3 content in the 2457 T and Sf14 genomes, the Wilcoxon Rank-Sum test was employed. At the group level, significance of GC values were analyzed via Kruskal-Wallis test with Dunn’s post-hoc test. The Wilcoxon Rank-Sum test was also used to analyze the significance of RSCU values of each codon for 2457 T and Sf14. A PERMANOVA test was also run on PCA data for RSCU values, which indicated a high level of significance and was followed up by a pairwise PERMANOVA with Bonferroni correction to assess the significance of RSCU differences between gene groups. The Wilcoxon Rank-Sum test was used to determine significance of tAI values at the genome level for Sf14 and host 2457 T, with effect size calculated using Cliff’s Delta test. At the gene group level, significance for tAI values was calculated using the Kruskal-Wallis test with post-hoc Dunn’s test. To assess effect size, Cliff’s Delta test was employed. To analyze differences in ∆G for phage tRNAs and host tRNAs, a paired t-test was run. The correlation between mutation position and ∆∆G values was calculated using the ANOVA test. Paired t-tests were run to identify significant differences between mutated and non-mutated tRNAs, as well as between consistent and inconsistent tRNAs. All data for all analyses are available in Supplementary Datasets 1–9.

## Results

### GC content of S. flexneri 2457 T and Shigella phage Sf14

**Fig. 1:**
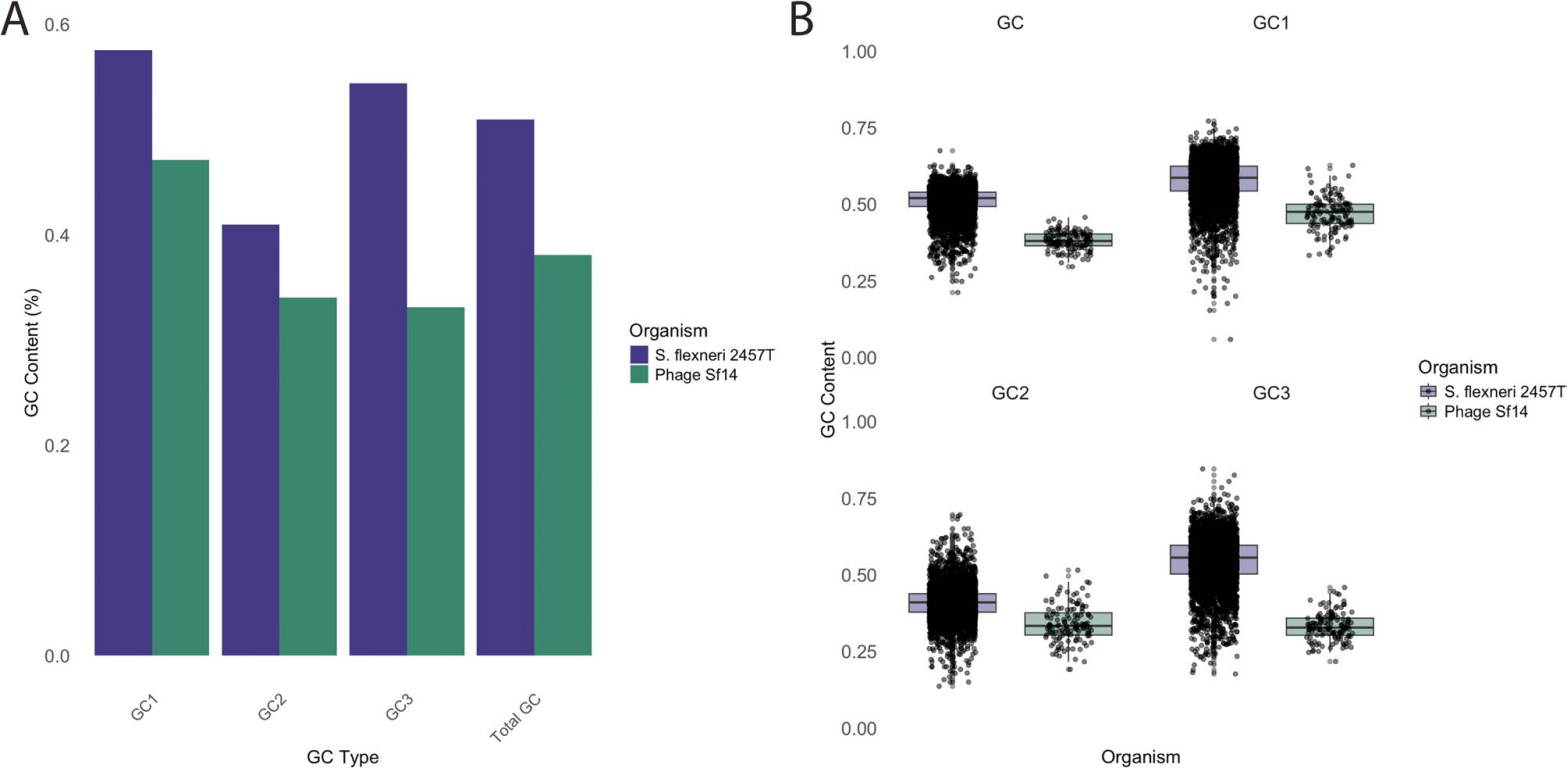
GC content of host and phage genomes overall. **A** Bar graph comparing total GC, GC1, GC2, and GC3 percentages in Sf14 and *S. flexneri* 2457 T genomes. **B** Box plots showing the distribution of GC content by gene for the Sf14 and 2457 T genomes. Total GC is in the top left, GC1 in the top right, GC2 in the bottom left, and GC3 in the bottom right of the figure

The total GC content of phage Sf14 and its host *S. flexneri* 2457 T was calculated, along with the GC content of each of the three codon positions, indicated as GC1, GC2, and GC3 for codon positions 1, 2, and 3, respectively [48]. In examining the genomes as a whole, the overall GC content of phage Sf14 is significantly lower than that of its host, with a total GC content of 38.09% compared to the 50.97% of its host strain 2457 T (p-value 3.58E-75). At the individual positions, GC1 content for Sf14 and 2457 T is 47.10% and 57.56% respectively (p-value 4.19E-51), GC2 content is 34.03% and 40.96% respectively (p-value 3.58E-34), and GC3 content is 33.12% and 54.40% respectively (p-value 1.11E-76). These values are listed in Table 1 and can be visualized in Fig. 1. Significance was determined using the Wilcoxon Rank-Sum test with continuity correction.

Since overall genome GC content may not be representative of individual genes in a genome, several groups of genes were also examined separately in our analyses. For *S. flexneri*, this included five sets of genes of involved in: 1) metabolism, 2) biosynthesis, 3) gene expression, 4) stress response/drug resistance, and 5) transport/miscellaneous. For Sf14, these were split into four groups: the temporal 1) early, 2) middle, and 3) late genes, plus the remaining 4) hypothetical genes. FASTA files of all genes in the groups and genomes are available in Supplementary Materials. The total GC%, along with the GC1, GC2, and GC3 percentages of these groups were then calculated and compared.

**Fig. 2:**
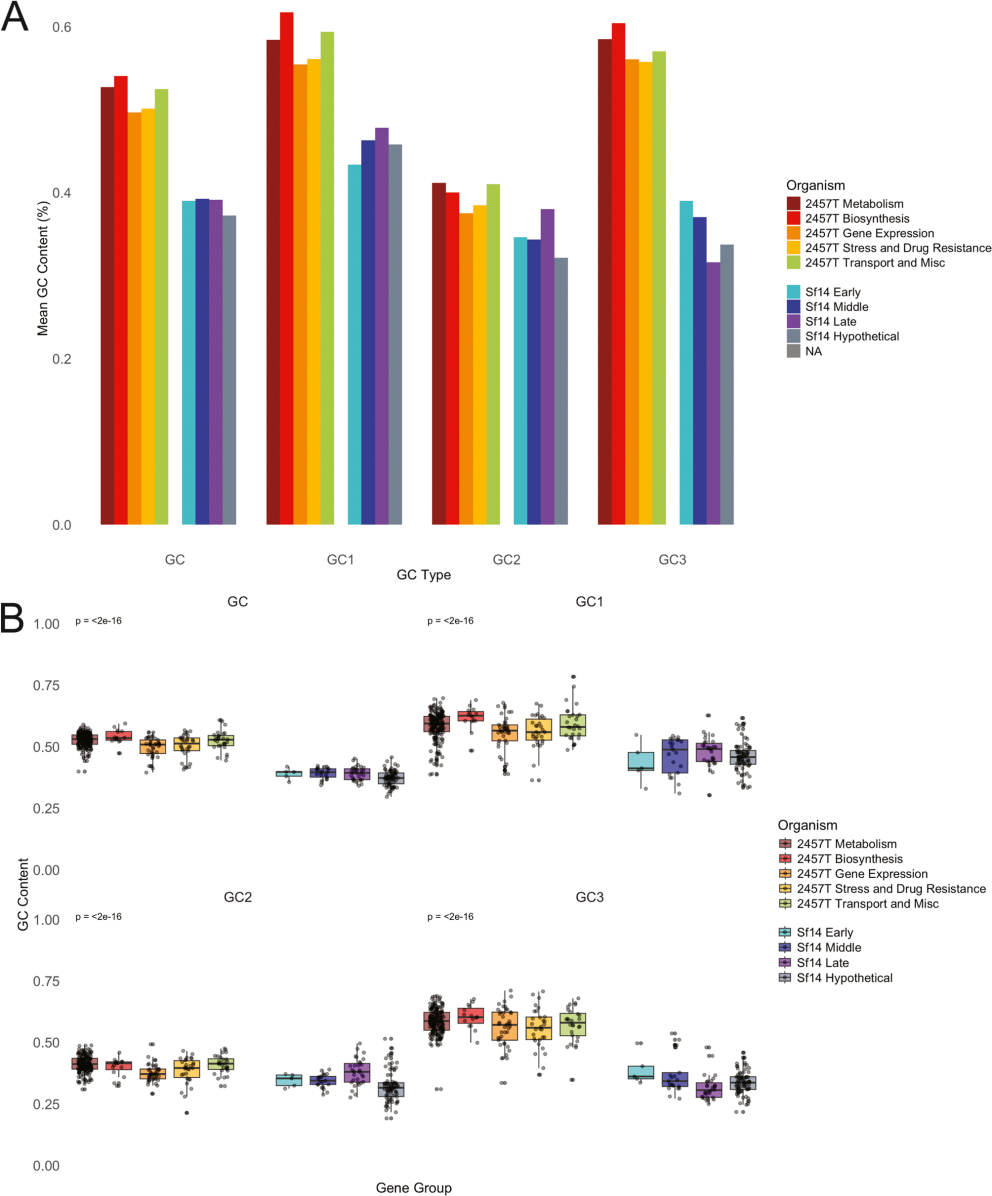
GC content of specific host and phage gene groups of interest. **A** Bar graph showing total GC, GC1, GC2, and GC3 percentages for all *S. flexneri* 2457 T and Sf14 gene groups. **B** Box plots of the distribution of total GC (top left), GC1 (top right), GC2 (bottom left), and GC3 (bottom right) for all 2457 T and Sf14 gene groups

Consistent with the genome level trends, the GC content of *S.* flexneri 2457 T genes were consistently higher than Sf14 genes. When examining individual codon positions, the Sf14 early genes had total GC and GC1 values significantly lower than the 2457 T metabolism, biosynthesis, and transport/miscellaneous genes; however, GC2 and GC3 values were not significantly different. Conversely, all GC values for Sf14 middle, late, and hypothetical genes – including total and position-specific GC values – were all significantly lower than those found in all 2457 T groups. Of these, the Sf14 hypothetical gene group had the lowest total GC content at 37.23%. In general, the lowest position-specific GC value for Sf14 genes was GC2, with 32.15% for hypothetical, 34.36% for middle, and 34.61% for early genes. The exception to this was the Sf14 late genes group, where GC3 was the lowest at 31.62%. Significance was determined using the Kruskal-Wallis rank sum test with Dunn’s post-hoc test. The total and position-specific GC values for all gene groups are presented in Table 1, with a comparison illustrated in Fig. 2. Data for all individual genes are available in Supplementary Dataset 1.

**Table 1:** GC content of Sf14 and its host *S. flexneri* 2457 T at the genome and gene group level

Organism/Group	Total GC%	GC1%	GC2%	GC3%
*S. flexneri* 2457 T Genome	50.975	57.562	40.962	54.401
Sf14 Genome	38.085	47.105	34.032	33.117
2457 T Metabolism	52.703	58.427	41.172	58.509
2457 T Biosynthesis	54.071	61.754	40.044	60.415
2457 T Gene Expression	49.681	55.445	37.531	56.066
2457 T Stress Drug Response	50.123	56.125	38.486	55.758
2457 T Transport/Misc.	52.490	59.405	41.032	57.032
Sf14 Early	39.006	43.389	34.609	39.020
Sf14 Middle	39.250	46.323	34.363	37.064
Sf14 Late	39.156	47.826	38.024	31.617
Sf14 Hypothetical	37.232	45.823	32.151	33.721

### Relative synonymous codon usage of Sf14 and host 2457T

**Fig. 3:**
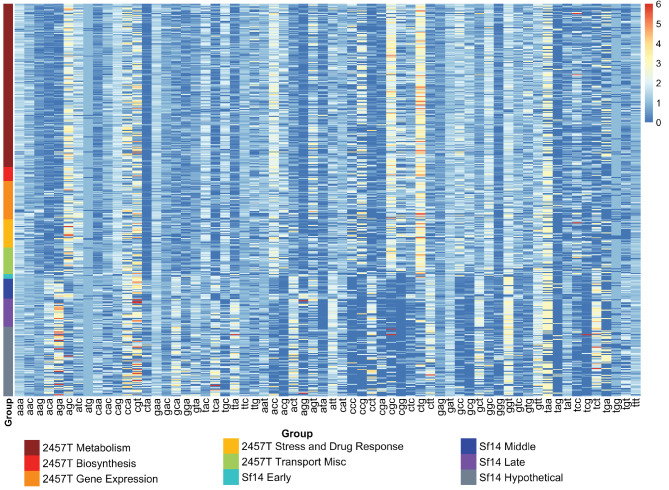
Heatmap of RSCU values for *S. flexneri* 2457 T and Sf14 gene groups. Genes are grouped by organism and split into their respective groups, as indicated by color on the y-axis. Colors correspond to the key on the top right. In the heatmap, blue represents low RSCU, with red representing high RSCU as indicated on the bottom right

Relative synonymous codon usage (RSCU) measures the relative use or bias of synonymous codons for an amino acid. The values for this metric range from 0 to the number of synonymous codons for a given amino acid. RSCU was calculated individually for each gene in Sf14 and its host *S. flexneri* 2457 T, then the Wilcoxon Rank-Sum test was used to determine the significance of the differences in the median RSCU values for all codons. Out of 61 amino acid-encoding codons, 51 have significantly different usage between Sf14 and *S. flexneri* 2457 T (p-values range from 0.0234 to 6.11E-64), with all data available in Supplementary Dataset 2 and visualized in Supplementary FigureS1. The genome-wide values can also be visualized as bar graphs in Supplementary FigureS2A and S3A**.** The exceptions are: Ile-ATA (p-value 0.0733) Arg-CGA and -CGT (p-values 0.1085 and 0.5285 respectively), Tyr-TAC and -TAT (p-values 0.5188 and 0.5610 respectively), Phe-TTC and -TTT (p-values 0.6833 and 0.7232 respectively), and Leu-TTG (p-value 0.9803). The RSCU of ATG (Met) and TGG (Trp) were equal to 1 for both genomes, as only one codon codes for each of those amino acids. RSCU values were also calculated for each gene within the four groups of Sf14 genes and the five groups of genes in 2457 T and compared via principal component analysis and pairwise PERMAN OVA. A heatmap to visualize the patterns of RSCU for these gene groups is presented in Fig. 3, with bar graphs for individual groups available in Supplementary Figure S2B-F and S3B-E. The STOP codons TGA, TAA, and TAG were excluded in the analysis. Particular interest was paid to codons for which Sf14 encodes a corresponding tRNA.

#### Sf14-encoded tRNAs and the RSCU of their corresponding codons

The 26 tRNAs encoded by Sf14 recognize 25 unique codons, with two tRNAs encoding acceptors for methionine (ATG). The program tRNAscan-SE 2.0 [33] was used to obtain isotype scores for all 26 tRNAs (Table 2). Of these, 13 unique tRNAs had anticodons that were inconsistent with the predicted isotype model: as examples, tRNA-Asn-GTT scored highest for Lys and tRNA-Lys-CTT scored highest for Cys. Since both tRNAs that recognize Met were inconsistent, this brings the total to 14 tRNAs. The biological significance of these inconsistencies is unknown but does not seem to be related to anticodon loop mutations, as discussed below (see “Mutations in Sf14-encoded tRNAs”).

**Table 2:** Isotype prediction scores for Sf14-encoded tRNAs. Glu, sec, val, and fMet were excluded from this table due to low scores for all predicted isotypes, but values for these can be found in Supplementary dataset 3. Colors correspond to score level, with red representing the highest score and yellow representing the lowest score. NH: no hit

Predicted isotype	Ala		Arg		Asn	Asp		Cys	Gln		Gly	His		Ile		Ile2	Leu		Lys	Met		Phe	Pro		Ser		Thr		Trp	Tyr						
Pro_TGG	57.2		62.4		38.2	5.6		27.1	41.5		40.6	43.9		64.5		47.4	34.9		57.9	50.6		60.5	73.1		1.5		65.5		10.9	No Hit						
Glu_TTC	2.2		56.8		9.3	48.6		58.4	54.6		32.2	33.9		53.5		40.7	52		47.7	14.1		31.6	34.2		18.2		40.9		26.8	16.2						
Met_CAT	16.6		29.9		10.3	−1.1		56.9	36.3		39.1	15.6		18.7		22.4	33.6		37.4	38.4		44.2	1.9		38.7		28.2		43	9.2						
Asn_GTT	11.8		32.2		47.9	NH		33.9	NH		26.7	10.3		19.7		38	5.1		52.7	24.9		26.4	19.8		29.7		25.6		2.1	26.3						
Tyr_GTA	−3.6		27.3		3.9	NH		23.1	−2.3		1.4	−7.2		4.9		32.9	22.1		31.3	NH		20.1	0.3		NH		8.9		18.5	60.3						
Asp_GTC	59.2		68.3		37.3	87		44.7	46.2		56.1	47.3		53.8		43.1	43.8		55.1	59.5		39.7	44.5		35.7		62.6		51.4	22.4						
Lys_TTT	16.0		33.7		25.4	3.2		50.8	29.9		34.4	43.5		44.4		53.6	34.6		38.4	37.3		35.2	17.9		41.6		32.3		42.1	37.2						
Met_CAT	22.9		36.3		16.5	6.8		63.8	36.3		45.9	15.6		24.7		28.6	40.1		44.5	45.2		50.6	8.8		44.8		34.5		43	16						
Ile_GAT	39.6		67.1		29.7	20.0		48.2	33.8		51.6	27.2		53.3		44.4	39.8		49.9	50.8		54.7	32.9		24.4		61.5		41.4	27.4						
Arg_TCT	46.4		82.6		53.3	50.8		57.9	49.6		48.6	44.7		46.6		73.7	47.1		59.5	32.2		63.7	28.1		27		52		75	28.5						
Ser_TGA	NH		15.1		NH	NH		8.7	7.6		7.0	0.7		NH		15.5	6.6		13.9	NH		1.4	NH		28.5		11.4		17.3	4						
Leu_TAG	34.5		60.0		27.3	7.6		41.7	38.2		27.7	31.2		28.9		65.7	67.4		63.4	30.6		48.3	13.8		23.8		41.5		43.6	34.7						
Lys_CTT	23.8		43.0		24.0	23.8		60.7	27.0		45.6	45.3		49		53.8	46.5		41.4	44.4		44.6	18.1		29.7		43.8		34.9	30.6						
Ala_TGC	58.2		44.1		NH	20.2		39.4	27.4		50.8	49.5		38.2		35.4	33.3		36	35.3		38.7	39.4		31.6		56.6		45.4	7.8						
Gly_TCC	46.8		50.3		15.7	29.1		36.3	28.9		61.4	25.3		26.3		37	26.9		43.3	19.3		39.3	18.4		18.1		42.4		55.4	6.5						
Thr_TGT	30.5		46.1		21.2	7.8		40.1	34.7		44.5	22.6		22.9		38.8	44		53.3	18.8		32.3	NH		19.2		50.7		36.8	34.4						
Val_TAC	27.1		47.1		−2.5	12.1		31.3	15.5		42.7	16.7		23.1		41.7	32.3		42.9	14.7		25.1	11.3		NH		40.9		29.6	1.8						
Leu_CAA	−0.3		34.4		20.0	2.4		27.2	16.7		18.8	22.3		21.1		35.1	49.1		36.1	25.7		30.1	1.4		15.1		30.6		15.5	14.8						
Arg_ACG	54.9		62.6		18.4	31.2		38.6	19.6		61.6	38.2		37		54.9	41.6		60.5	32.7		60	27.7		23.2		52.8		53.1	16.6						
Gln_TTG	16.3		39.8		−6.3	18.6		42.3	31.4		43.6	30.6		26.8		42	21.1		41.1	18.1		35.1	34.6		28.1		37.7		39.5	17.1						
Leu_TAA	7.3		43.0		29.4	NH		38.4	36		19.9	25.7		19.8		23.9	60.2		54.2	24.9		45.1	NH		21.5		37.4		32.1	32.6						
Gln_CTG	40.8		56.1		28.4	18.7		52.4	55.5		46.6	30.3		47.4		50.5	38.7		40.1	32.4		31.4	34.8		22.7		44.7		57.9	24.7						
His_GTG	NH		40.9		NH	5.9		21.7	−3.2		24.7	31.6		11.6		41.3	25.5		6.8	0.4		13.9	−0.6		4.2		36.1		28.8	NH						
Phe_GAA	32.4		32.2		−7.5	7.7		30.5	4.5		21.7	6.1		30.7		34.1	NH		29	23.8		13.8	4.5		5.8		31		6.6	−7.3						
Ser_GCT	8.0		27.1		−11.6	NH		42.4	NH		33.1	NH		7.3		12.3	35		23.1	8.4		22.9	16.8		38.8		36.1		35	37.7						
Cys_GCA	22.2		37.4		−2.3	34.8		46.2	12.5		35.1	32.1		3		27.4	40		25.3	18.1		23.1	11.8		38.2		28.8		32.1	8.2						

The RSCU values for codons for which Sf14 encodes a corresponding tRNA decoder were specifically of interest. The median RSCU values for 20 of the 25 codons for which Sf14 encodes an isodecoder were significantly different in the phage compared to its host, with some codons being used less frequently by Sf14 (TGC, p-value 3.99E-11; GAA, p-value 0.0114; ATC, p-value 5.70E-09; AAA, p-value 1.87E-22; CTA, p-value 1.48E-17; CAG, p-value 2.30E-20; AGC, p-value 4.29E-26). Others were used more frequently in Sf14 than in the host strain 2457 T (GCA, p-value 9.76E-36; GGA, p-value 8.7E-4; CAC, p-value 0.0234; AAG, p-value 7.63E-23; TTA, p-value 2.24E-26; CTA, p-value1.48E-17; CCA, p-value 1.76E-24; CAA, p-value 2.62E-20; AGA, p-value 3.46E-30; TCA, p-value 1.73E-20; ACA, p-value 1.74E-33; GTA, p-value 1.48E-27). These data, along with whether the anticodons were consistent or inconsistent with the predicted isotype model, are summarized in Table 3.

**Table 3:** Codon frequency (%) and mean and median RSCU for Sf14-encoded tRNAs in *S. flexneri* 2457 T and *Shigella* phage Sf14

				Frequency(%)		Mean RSCU		Median RSCU			
Consistency	AC	AA	Codon	2457T	Sf14	2457T	Sf14	2457T	Sf14	*p*-value	α
Consistent	TGC	Ala	GCA	2.197	3.062	0.995	1.734	**0.842**	**1.714**	0.000	***
Consistent	GCA	Cys	TGC	2.115	0.436	1.135	0.397	**1.063**	**0.667**	0.000	***
Consistent	GTC	Asp	GAC	1.383	2.152	0.785	0.661	**0.741**	**0.667**	0.014	*
Inconsistent	TTC	Glu	GAA	2.507	4.338	1.311	1.301	**1.375**	**1.333**	0.011	*
Inconsistent	GAA	Phe	TTC	1.508	1.907	0.868	0.802	0.800	0.857	0.683	
Consistent	TCC	Gly	GGA	1.498	1.035	0.849	0.769	**0.444**	**0.667**	0.001	***
Inconsistent	GTG	His	CAC	1.284	0.969	0.936	0.971	**0.833**	**1.000**	0.023	*
Inconsistent	GAT	Ile	ATC	1.786	1.965	1.113	0.962	**1.200**	**0.912**	0.000	***
Inconsistent	CTT	Lys	AAG	1.545	3.393	0.747	0.795	**0.462**	**0.778**	0.000	***
Inconsistent	TTT	Lys	AAA	2.777	4.533	1.253	1.205	**1.539**	**1.222**	0.000	***
Consistent	CAA	Leu	TTG	1.633	1.202	1.178	0.741	0.692	0.750	0.980	
Consistent	TAA	Leu	TTA	1.506	1.930	1.057	1.629	**0.706**	**1.563**	0.000	***
Consistent	TAG	Leu	CTA	0.700	0.825	0.429	0.694	**0.164**	**0.633**	0.000	***
Inconsistent	CAT	Met	ATG	2.061	2.922	1.000	1.000	1.000	1.000	NA	
Inconsistent	GTT	Asn	AAC	1.961	2.584	1.008	0.957	**1.000**	**1.000**	0.012	*
Consistent	TGG	Pro	CCA	1.333	1.412	1.262	1.873	**0.714**	**2.000**	0.000	***
Inconsistent	CTG	Gln	CAG	2.114	1.724	1.180	0.885	**1.333**	**1.000**	0.000	***
Inconsistent	TTG	Gln	CAA	1.642	1.891	0.820	1.115	**0.667**	**1.000**	0.000	***
Consistent	TCT	Arg	AGA	1.153	1.412	0.753	2.114	**0.000**	**2.000**	0.000	***
Consistent	ACG	Arg	CGT	1.839	1.607	0.986	2.351	2.000	2.000	0.529	
Inconsistent	GCT	Ser	AGC	1.721	0.794	1.221	0.778	**1.565**	**0.652**	0.000	***
Consistent	TGA	Ser	TCA	1.491	1.751	1.269	1.554	**0.667**	**1.539**	0.000	***
Inconsistent	TGT	Thr	ACA	1.239	2.521	0.925	1.505	**0.500**	**1.455**	0.000	***
Inconsistent	TAC	Val	GTA	1.126	2.187	0.830	1.242	**0.588**	**1.333**	0.000	***
Consistent	GTA	Tyr	TAC	1.251	1.875	0.914	0.850	0.870	0.817		

tRNAs showing inconsistency between the predicted model and actual isotype are highlighted in orange. Bolded median RSCU values are those for which Sf14 and host 2457 T have significantly different codon usage

*AC* Anticodon, *AA *Amino acid, *RSCU* Relative synonymous codon usage

α represents the significance level, where * ≤0.05, ** ≤0.005, *** ≤ 0.001

#### Principal component analysis of RSCU values

**Fig. 4:**
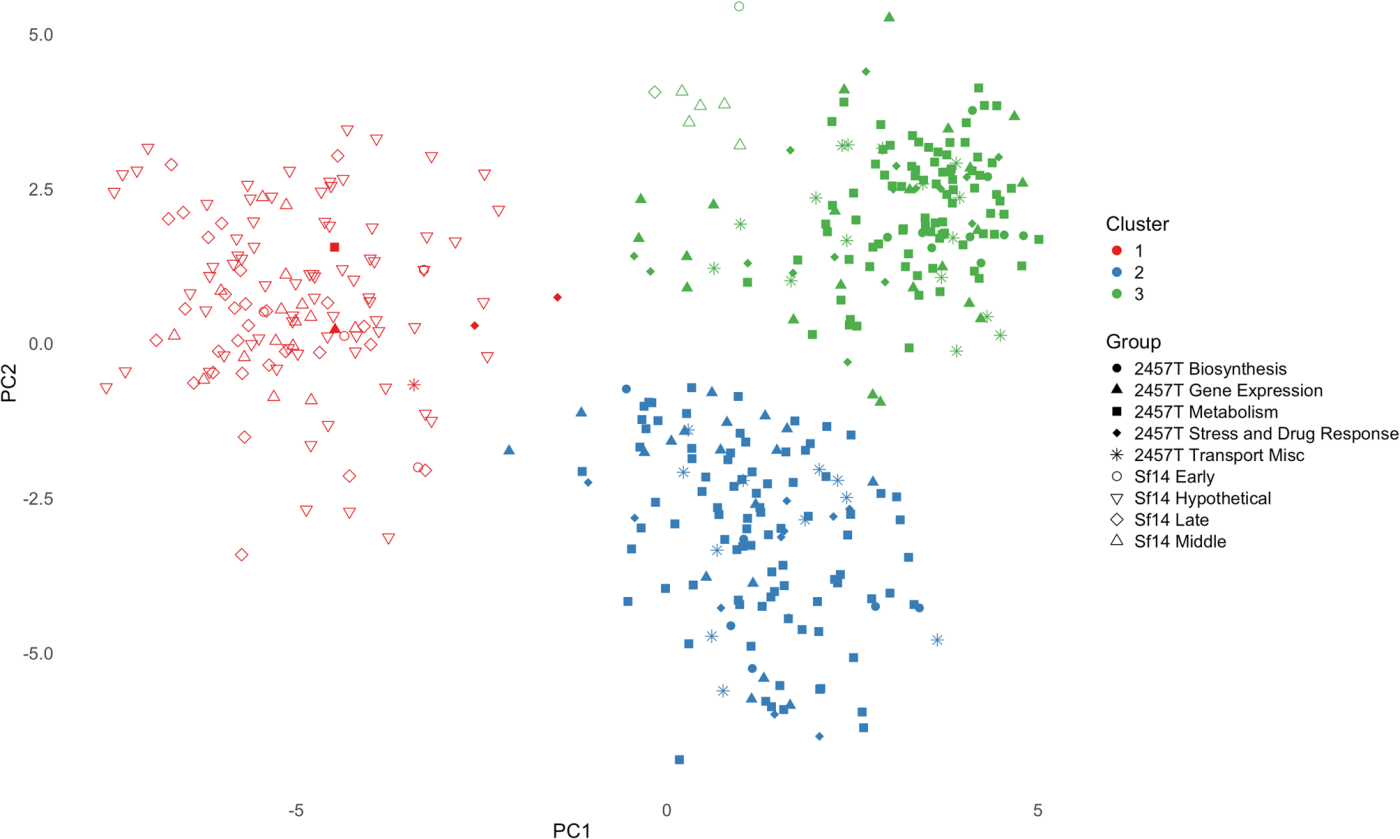
PCA plot of RSCU for *S. flexneri* 2457 T and Sf14 gene groups of interest. Genes are assigned a color and symbol based on their group, as indicated in the key on the right. An alternative labeled version of this figure is available in Supplementary Figure S4, with a list of genes provided in Supplementary Table S2, tab of PCA_Genes_by_Cluster

A principal component analysis was conducted in R, the results of which can be seen in Fig. 4. Significance was determined via PERMANOVA and pairwise PERMANOVA. There were significant differences between the groups of genes investigated, with an F statistic of 15.111 and a p-value of < 0.001.A pairwise PERMANOVA was run to further investigate the relationships between the groups of genes: for *S. flexneri* 2457 T, genes involved in metabolism, biosynthesis, gene expression, stress response/drug resistance, and transport/miscellaneous; for Sf14, genes expressed during the early, middle, and late stages of infection, along with hypothetical genes. These data are presented in Table 4.

**Table 4:** Pairwise PERMANOVA of 2457 T and Sf14 gene groups

Paired Groups		DF	SS	R2	F	Pr(> F)	α
2457 T Metabolism	2457 T Biosynthesis	1	23.200	0.005	0.953	0.419	
**2457 T Metabolism**	** 2457 T Gene Expression**	**1**	**66.600**	**0.012**	**2.627**	**0.010**	*
**2457 T Metabolism**	** 2457 T Stress/Drug**	**1**	**59.000**	**0.011**	**2.282**	**0.020**	*
2457 T Metabolism	2457 T Transport/Misc.	1	18.600	0.004	0.740	0.689	
**2457 T Metabolism**	**Sf14 Early**	**1**	**122.500**	**0.027**	**4.945**	**0.001**	***
**2457 T Metabolism**	**Sf14 Middle**	**1**	**656.400**	**0.121**	**26.401**	**0.001**	***
**2457 T Metabolism**	**Sf14 Late**	**1**	**1264.900**	**0.201**	**50.569**	**0.001**	***
**2457 T Metabolism**	**Sf14 Hypothetical**	**1**	**2031.200**	**0.206**	**63.568**	**0.001**	***
2457 T Biosynthesis	2457 T Gene Expression	1	36.200	0.024	1.347	0.155	
2457 T Biosynthesis	2457 T Stress/Drug	1	28.030	0.022	0.945	0.449	
2457 T Biosynthesis	2457 T Transport/Misc.	1	21.000	0.019	0.806	0.605	
**2457 T Biosynthesis**	**Sf14 Early**	**1**	**108.440**	**0.199**	**4.473**	**0.002**	**
**2457 T Biosynthesis**	**Sf14 Middle**	**1**	**359.100**	**0.297**	**14.348**	**0.001**	***
**2457 T Biosynthesis**	**Sf14 Late**	**1**	**553.420**	**0.334**	**21.542**	**0.001**	***
**2457 T Biosynthesis**	**Sf14 Hypothetical**	**1**	**581.600**	**0.130**	**12.954**	**0.001**	***
2457 T Gene Expression	2457 T Stress/Drug	1	40.250	0.019	1.305	0.167	
2457 T Gene Expression	2457 T Transport/Misc.	1	40.500	0.021	1.412	0.126	
**2457 T Gene Expression**	**Sf14 Early**	**1**	**90.330**	**0.065**	**3.082**	**0.005**	**
**2457 T Gene Expression**	**Sf14 Middle**	**1**	**388.200**	**0.186**	**13.667**	**0.001**	***
**2457 T Gene Expression**	**Sf14 Late**	**1**	**708.830**	**0.266**	**24.980**	**0.001**	***
**2457 T Gene Expression**	**Sf14 Hypothetical**	**1**	**856.500**	**0.153**	**20.337**	**0.001**	***
2457 T Stress/Drug	2457 T Transport/Misc.	1	24.640	0.014	0.790	0.672	
**2457 T Stress/Drug**	**Sf14 Early**	**1**	**91.310**	**0.076**	**2.707**	**0.008**	**
**2457 T Stress/Drug**	**Sf14 Middle**	**1**	**380.220**	**0.199**	**12.193**	**0.001**	***
**2457 T Stress/Drug**	**Sf14 Late**	**1**	**658.660**	**0.270**	**21.442**	**0.001**	***
**2457 T Stress/Drug**	**Sf14 Hypothetical**	**1**	**752.100**	**0.141**	**16.740**	**0.001**	***
**2457 T Transport/Misc.**	**Sf14 Early**	**1**	**100.320**	**0.100**	**3.429**	**0.002**	**
**2457 T Transport/Misc.**	**Sf14 Middle**	**1**	**403.910**	**0.234**	**14.366**	**0.001**	***
**2457 T Transport/Misc.**	**Sf14 Late**	**1**	**688.410**	**0.304**	**24.474**	**0.001**	***
**2457 T Transport/Misc.**	**Sf14 Hypothetical**	**1**	**772.100**	**0.150**	**17.642**	**0.001**	***
Sf14 Early	Sf14 Middle	1	32.660	0.045	1.138	0.278	
**Sf14 Early**	**Sf14 Late**	**1**	**57.660**	**0.058**	**2.019**	**0.039**	*
Sf14 Early	Sf14 Hypothetical	1	48.800	0.013	1.004	0.447	
**Sf14 Middle**	**Sf14 Late**	**1**	**54.740**	**0.039**	**1.976**	**0.023**	*
Sf14 Middle	Sf14 Hypothetical	1	42.700	0.010	0.956	0.477	
**Sf14 Late**	**Sf14 Hypothetical**	**1**	**75.600**	**0.017**	**1.747**	**0.021**	*

Bolded rows are those for which there is a significant difference in RSCU between the groups compared

*SS *Sum of squares, *DF* Degrees of freedom, *Stress/Drug* Stress Response and Drug Resistance

α represents the significance level, where * ≤0.05, ** ≤0.005, *** ≤ 0.001

Results consistently show significant differences in RSCU between all Sf14 gene groups and the RSCU values of the host *S. flexneri* 2457 T gene groups. There were also significant differences in codon usage between an organism’s gene groups. For 2457 T, these were namely between metabolism genes and the gene expression and stress response/drug resistance gene groups. For Sf14, there were significant differences in the codon usage of the late genes compared to the early, middle, and hypothetical genes, indicating the codon usage of the late-expressed structural genes differs from that of genes expressed in the earlier stages of infection.

#### Sf14-encoded tRNA-Lys-CTT

In comparing the tRNAs each encoded by phage and host, we determined that only Sf14 encodes a corresponding tRNA for the lysine codon AAG, while *S. flexneri* 2457 T does not. To investigate this further, analyses were conducted specifically on the AAG codon/tRNA-Lys-CTT. For Sf14, 41 out of 131 (31.30%) coding sequences have an RSCU ≥ 1 for codon AAG, compared to only 406 of 4495 (9.03%) coding sequences in *S. flexneri* 2457T. The median RSCU for codon AAG in Sf14 is 0.778, compared to the median 2457 T RSCU of 0.462 (p-value 7.63E-23). When investigating specific groups of Sf14-encoded genes, it was found that 12 out of 30 annotated late/structural genes have an RSCU of ≥ 1 for codon AAG, compared to 2 out of 5 early genes, 6 out of 21 middle genes, and 19 out of 74 hypothetical genes. This may indicate a need for the lysine tRNA-CTT to maintain or increase translation efficiency during later stages of Sf14 infection. Complete RSCU data are available in Supplementary Dataset 3.

### tRNA adaptation index for S. flexneri 2457 T and Shigella phage Sf14

Once RSCU was determined, we next calculated the tRNA adaptation index (tAI) of the *S. flexneri* 2457 T and Sf14 genomes, along with all investigated genes of interest. This was done using an R script based on the python script from [25]. As a genome-wide overview, the tAI results for each gene in the 2457 T and Sf14 genomes are available in Supplementary Figures S5 and S6. A Wilcoxon Rank-Sum test was used to determine if differences in tAI existed between the Sf14 and *S. flexneri* 2457 T genomes. This analysis indicates that the differences in tAI at the genome level are indeed significant, with *S. flexneri* 2457 T having a median and mean tAI of 0.3 and 0.309, respectively, and Sf14 having a median and mean tAI of 0.135 and 0.281, respectively (p-value 0.005). Cliff’s Delta test for effect size was performed next, which revealed a delta estimate of 0.143 (95% CI 0.023, 0.260), indicating that the biological effect of this difference is negligible.

**Fig. 5:**
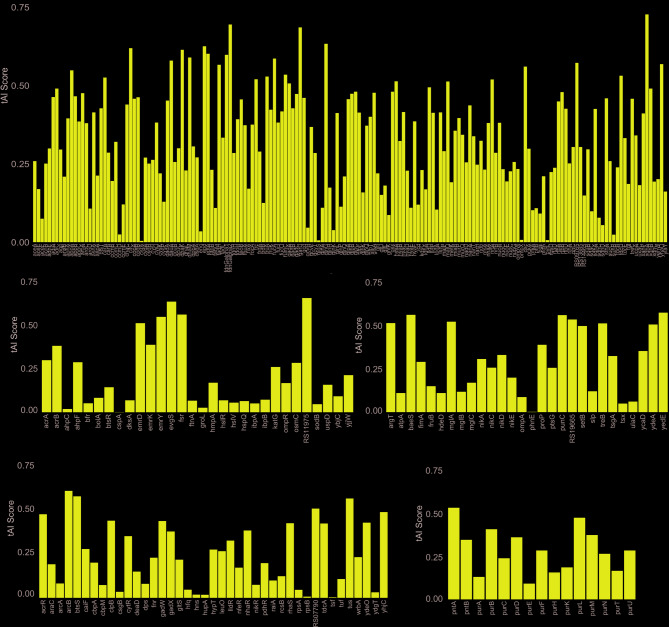
*S. flexneri * 2457 T gene group tAI values. Bar graphs depicting tAI scores for *S. flexneri* 2457 T genes in the groups of: metabolism, gene expression, biosynthesis, stress response and drug resistance, and transport and miscellaneous

**Fig. 6:**
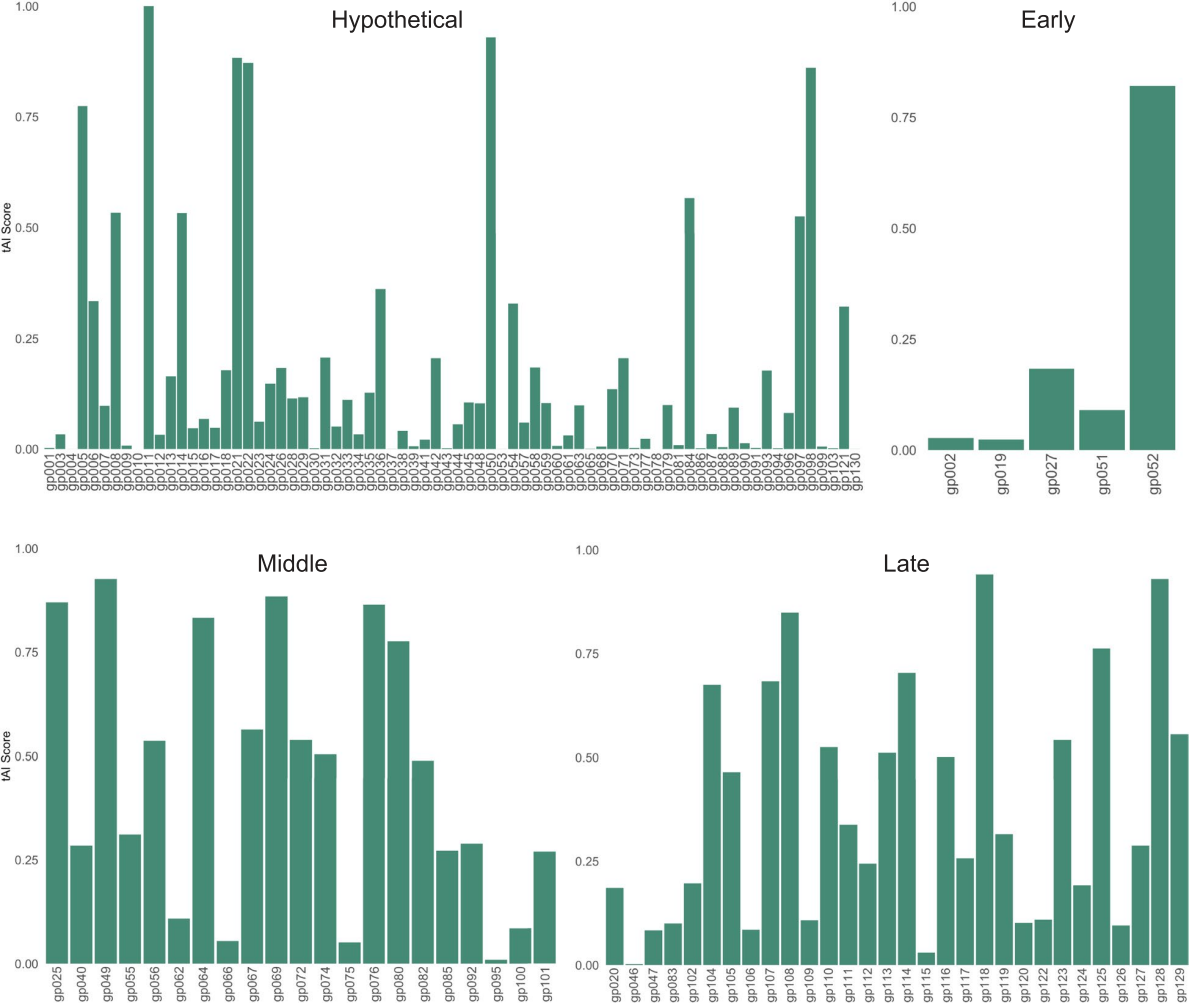
Phage Sf14 gene group tAI values. Bar graphs depicting the tAI values for Sf14 genes in hypothetical, early, middle, and late gene groups

After establishing that differences between the genomes exist, tAI values for the specific gene groups were investigated. The same script as above was used to calculate the tAI of 2457 T and Sf14 gene groups, which are shown in Figs. 5 and 6. A statistical summary of the tAI values, including sample size, mean tAI, median tAI, and standard deviation, is also presented in Table 5. Bar plots of the group tAI data are available in Supplementary Figures S5 and S6, with all values for individual genes at the genome and group level available in Supplementary Dataset 4. High tAI values are associated with greater codon usage bias, increased efficiency of translation, and higher levels of translation [26]. This is consistent with our data for *S. flexneri* 2457 T, as the mean and median tAI values are higher for genes known to have high levels of expression, such as those involved in metabolism and transport. For Sf14, we anticipated that the late genes of Sf14 would have a high tAI since these are produced at high levels for virion assembly to occur. The mean tAI for these genes was indeed higher than the early and hypothetical genes at 0.379, with a median tAI of 0.302 (standard deviation 0.285). Interestingly, the genes expressed during the middle stages of infection – those involved in DNA replication and repair – have even higher tAI values, with a mean of 0.453 and a median of 0.489 (standard deviation 0.312). This difference, however, is not significant between groups.

**Table 5:** Statistical summary of tAI values for gene groups of interest

Gene Group	*n*	mean	median	std dev
Metabolism	172	0.325	0.313	0.166
Biosynthesis	15	0.279	0.277	0.126
Gene Expression	41	0.244	0.216	0.182
Stress/Drug	30	0.214	0.150	0.199
Transport/Misc	28	0.302	0.298	0.187
Early	5	0.229	0.090	0.337
Middle	21	0.453	0.489	0.312
Late	30	0.379	0.302	0.285
Hypothetical	74	0.170	0.065	0.254

**Fig. 7:**
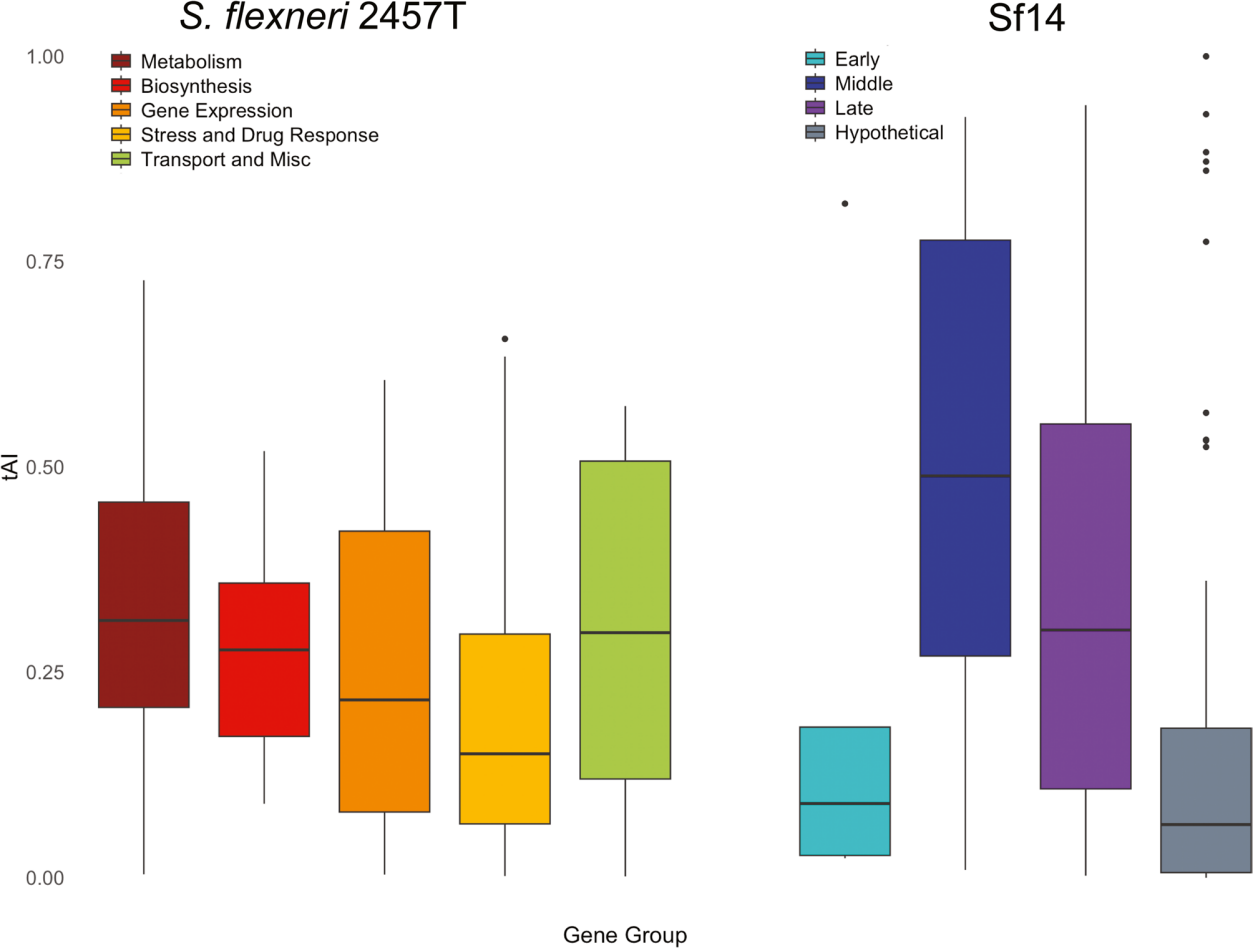
tAI distribution by gene group. Boxplot depicting tAI distribution for all gene groups from *S. flexneri* 2457 T (left) and Sf14 (right). Groups are colored according to the key under the organism name. Outliers are indicated by black circles

Unlike the RSCU comparison, there were few significant differences between Sf14 and 2457 T gene groups (Fig. 7). Host gene groups tend to be highly expressed, with the exception of stress response/drug resistance genes, which may explain this observation. The only differences in tAI were between the Sf14 hypothetical genes and the *S. flexneri* 2457 T metabolism genes (p-value 3.41E-11) and transport/miscellaneous genes (p-value 2.71E-3). Within the Sf14 genome, hypothetical genes also had significantly lower tAI values than those involved in both middle and late stages of infection (p-value 1.25E-5 and 7.77E-5, respectively). The reason for this is unknown, as the hypothetical genes are not well-characterized and conclusions on their role in infection cannot be drawn. Full statistical analyses data are also available in Supplementary Dataset 4.

#### High tAI genes in the Sf14 genome

Next, Sf14-encoded genes with a high tAI (≥ 0.5) were investigated further. Of the 131 Sf14 genes, 37 had a tAI ≥ 0.5, with 25 in the hypothetical gene group, 6 in the middle gene group, and 6 in the late gene group. Middle genes with high tAI included those for the DNA-directed DNA polymerase and other enzymes involved in DNA synthesis. The late genes with highest tAI values were components of the baseplate and tail.

Because Sf14 encodes a tRNA-Lys-CTT while the host *S. flexneri* 2457 T does not, how the RSCU of lysine codons relate to tAI values was of interest. We hypothesized that phage genes with high tAI values would also have an RSCU > 1 for Lysine-AAG; however, this was not the case. As listed in Table 6, the RSCU values for codons AAA and AAG in genes with high tAI are similar, with only one middle gene (gp085, a DNA-directed DNA polymerase) and one late gene (gp105, a tail fiber protein) having an RSCU > 1 for Lys-AAG. There were also four hypothetical genes with an RSCU > 1 for codon AAG (gp5, gp50, gp53, and gp90), but because these genes are hypothetical, conclusions about them cannot be drawn. Thus, differences in synonymous codon usage for lysine do not necessarily correlate with higher tAI levels. All data used in this analysis for both phage and host genes are available in Supplementary Dataset 5.

**Table 6:** tAI values and lysine RSCU for High-tAI genes encoded by Sf14

Locus Tag	tAI	AAA	AAG	Group	Gene Product
Sf14_gp011	1.000	0.833	1.167	Hypothetical	
Sf14_gp039	0.941	1.429	0.571	Hypothetical	
Sf14_gp107	0.929	1.579	0.421	Late	baseplate protein
Sf14_gp029	0.929	1.333	0.667	Hypothetical	
Sf14_gp108	0.926	1.000	1.000	Late	baseplate wedge subunit
Sf14_gp088	0.885	1.500	0.500	Hypothetical	
Sf14_gp021	0.883	1.091	0.909	Hypothetical	
Sf14_gp022	0.872	1.538	0.462	Hypothetical	
Sf14_gp025	0.870	1.091	0.909	Middle	RNA-binding protein
Sf14_gp081	0.864	1.000	1.000	Hypothetical	
Sf14_gp059	0.861	1.067	0.933	Hypothetical	
Sf14_gp049	0.849	1.143	0.857	Middle	polynucleotide kinase
Sf14_gp093	0.833	1.200	0.800	Hypothetical	
**Sf14_gp105**	**0.821**	**0.545**	**1.455**	**Late**	**tail fiber protein**
Sf14_gp072	0.783	1.500	0.500	Middle	MazG-like pyrophosphatase
Sf14_gp077	0.776	1.032	0.968	Hypothetical	
**Sf14_gp005**	**0.774**	**0.593**	**1.407**	**Hypothetical**	
Sf14_gp032	0.762	1.111	0.889	Hypothetical	
Sf14_gp043	0.703	1.091	0.909	Hypothetical	
**Sf14_gp050**	**0.683**	**0.815**	**1.185**	**Hypothetical**	
**Sf14_gp053**	**0.675**	**0.800**	**1.200**	**Hypothetical**	
Sf14_gp020	0.594	1.091	0.909	Late	capsid decoration protein
Sf14_gp073	0.566	1.200	0.800	Hypothetical	
**Sf14_gp090**	**0.564**	**0.857**	**1.143**	**Hypothetical**	
Sf14_gp028	0.556	1.600	0.400	Hypothetical	
Sf14_gp056	0.548	1.300	0.700	Middle	ribose-phosphate pyrophosphokinase
Sf14_gp034	0.542	1.500	0.500	Hypothetical	
**Sf14_gp085**	**0.539**	**0.979**	**1.021**	**Middle**	**DNA-directed DNA polymerase**
Sf14_gp101	0.536	1.200	0.800	Middle	thymidylate synthase
Sf14_gp008	0.533	1.333	0.667	Hypothetical	
Sf14_gp014	0.532	1.059	0.941	Hypothetical	
Sf14_gp047	0.525	1.429	0.571	Late	Rz-like spanin (inner membrane)
Sf14_gp060	0.524	1.273	0.727	Hypothetical	
Sf14_gp057	0.516	1.667	0.333	Hypothetical	
Sf14_gp044	0.511	1.333	0.667	Hypothetical	
Sf14_gp083	0.504	1.333	0.667	Late	minor tail protein
Sf14_gp041	0.501	1.600	0.400	Hypothetical	

Bolded rows are those for which the AAG codon is used more frequently than AAA

### Assessing host and phage tAI with host, phage, or combined tRNA pools

**Fig. 8:**
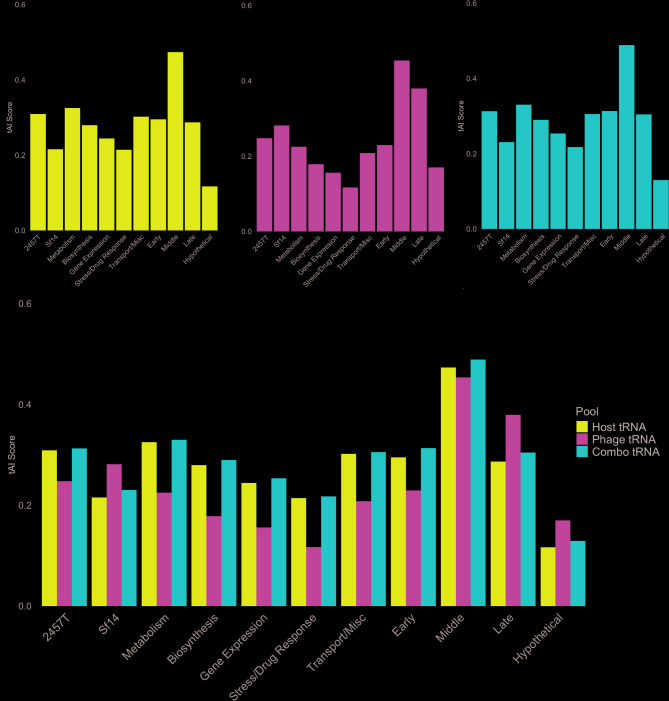
Comparison of tAI calculated with different tRNA pools. Bar graphs depicting the tAI values for Sf14 and *S. flexneri* 2457 T genomes and all gene groups calculated using the (**A**) host tRNA pool, (**B**) phage tRNA pool, or (**C**) the combined tRNA pool. **D** Shows the average tAI for all tRNA pools for Sf14 and 2457 T genomes and all gene groups

The above results report on the tAI values when the phage and host are relying on their respective tRNA pools; however, since infection does not proceed in a vacuum, each of these on their own is not necessarily reflective of the total available tRNA pool in the cell. To compare the effect of available tRNA pool on the efficiency of translation, all three tRNA pools were examined: *S. flexneri* 2457 T host tRNAs only (“host tRNA pool” with 100 tRNAs), Sf14 phage tRNAs only (“phage tRNA pool” with 26 tRNAs), and a combination library consisting of both 2457 T and Sf14 tRNAs (“combined tRNA pool” with 126 tRNAs). The first of these assumes either no infection or no expression of phage tRNAs, as in early infection; the second assumes that host tRNAs have been degraded or are inactive, which may occur during late infection; and the third assumes both sets of tRNA are present, which may occur in middle and/or late infection stages. The differences between these groups were calculated and analyzed with either the paired t-test or the Wilcoxon Rank-Sum test, depending on the distribution of the data. The average tAI values for all sets of tRNAs are summarized in Table 7, with complete data available in Supplementary Datasets 6–8. Comparisons across all tRNA pools can also be found in Fig. 8.

**Table 7:** Average tAI values for host-only, phage-only, and combined tRNA pools

Genome or Group	Average tAI when using…		
	Host tRNA	Phage tRNA	Both tRNAs
2457T	0.309	0.247	0.313
2457 T Genes of Interest	0.297	0.200	0.303
2457 T Metabolism	0.325	0.225	0.330
2457 T Biosynthesis	0.279	0.178	0.289
2457 T Gene Expression	0.244	0.156	0.253
2457 T Stress/Drug	0.214	0.117	0.217
2457 T Transport/Misc	0.302	0.208	0.305
Sf14	0.215	0.281	0.230
Sf14 early	0.295	0.229	0.313
Sf14 mid	0.473	0.453	0.489
Sf14 late	0.287	0.379	0.304
Sf14 hypothetical	0.117	0.170	0.129

#### tAI comparison of host tRNA pool to phage tRNA pool

First, we calculated and compared the host’s tAI values using the host tRNA pool or the phage tRNA pool. *S. flexneri* 2457 T overall, along with most of its gene groups, had significantly higher tAI values when using its own tRNAs compared to those calculated with phage tRNAs. The genome-wide average was 0.309 vs. 0.247 (p-value 2.20E-16), respectively, as determined by the Wilcoxon Rank-Sum test. The differences in average tAI using host or phage tRNA pools were also significant for the majority of *S. flexneri* 2457 T groups. Based on the Wilcoxon Rank-Sum test, differences for host or phage tRNA pools for metabolism genes were 0.325 vs. 0.225 (p-value 2.20E-16); and for gene expression genes, 0.244 vs. 0.156 (p-value 1.93E-6). Differences for genes involved in stress response and drug resistance (0.214 vs. 0.117, p-value 3.327E-4), and transport and miscellaneous genes (0.302 vs. 0.208, p-value 1.79E-5) were calculated using the paired t-test. Perhaps unsurprisingly, this suggests *S. flexneri* 2457 T genes are well-adapted to its own tRNA pool and not to the phage tRNAs. The genes associated with biosynthesis, which showed no significant difference between pools, were the only exception.

Next, tAI values were calculated for Sf14 genes using the host tRNA pool or the phage tRNA pool. Comparisons using the Wilcoxon Rank-Sum test revealed a significant difference in the average tAI of 0.215 vs. 0.281 (p-value 8.68E05), with the phage tRNA pool resulting in a significantly higher average tAI. However, this is not evenly distributed across the gene groups. The Sf14-encoded early (0.295 vs. 0.229) or middle (0.473 vs. 0.453) genes show a slight bias towards the host tRNA pool, but the difference between host and phage tRNA pools are not significant. Conversely, the late genes exhibit a preference for the phage tRNA pool over that of the host that is significant (0.287 vs. 0.379, p-value 0.013), indicating that phage-encoded tRNAs are used preferentially during the later stages of infection. Similarly, the Sf14-encoded hypothetical genes show an overall preference for the phage tRNA pool, regardless of their location in the genome. When comparing tAI values calculated using the host tRNA pool compared to those calculated using the phage tRNA pool, the average tAI values for hypothetical genes are significantly higher when using the phage tRNA pool only (0.117 vs. 0.170, p-value 0.0012). Average values for both genomes and all gene groups using the host and phage tRNA pools are depicted in Fig. 8A-B, respectively.

#### tAI comparison of host tRNA pool to combined tRNA pool

During infection, the phage tRNAs are theoretically adding to the host tRNA pool for some portion of the infection cycle. If the phage tRNAs are supplementing the available host tRNAs, perhaps redirecting resources to a specific set of genes, we hypothesized the combined tRNA pool may have higher tAI values than those calculated using only the host tRNA pool. To investigate this, we compared tAI calculated with host-only or combined tRNA pools and found that *S. flexneri* 2457 T overall had an average tAI of 0.309 and 0.313 respectively (p-value 2.20E-16). This is also reflected in the 2457 T gene groups that were examined, with the average tAI for the host tRNA pool compared to that of the combined tRNA pool being significantly higher for metabolism genes (0.325 vs. 0.330, p-value 1.47E-11), genes involved in gene expression (0.244 vs. 0.253, p-value 1.27E-9), and genes involved in stress response and drug resistance (0.214 vs. 0.217, p-value 0.0145). However, there was no significant difference in tAI values for the 2457 T genes involved in biosynthesis or transport/miscellaneous activities within the cell. These comparisons are shown in Fig. 8A and C.

Next, we calculated the tAI values for Sf14 using the host-only or the combined tRNA pool. Similar to the host, Sf14 genes in general had higher tAI values when using a combined tRNA pool. The Sf14 genome reports average tAI values of 0.215 when using the host tRNAs, and an average tAI of 0.230 when using a combined tRNA pool (p-value 2.20E-16). This was the case for all Sf14 gene groups, with the average tAI showing a significant increase when using the combined tRNA pool. In comparing host-only to combined pools, the average tAI for early genes were 0.295 vs. 0.313 respectively (p-value 1.59E-4), middle genes were 0.473 vs. 0.489 (p-value 1.05E-4), late genes were 0.287 vs. 0.304 (p-value 1.19E-6), and hypothetical genes were 0.117 vs. 0.129 (p-value 1.19E-13). Due to the results in the previous section, we speculated that this is due specifically to the addition of the Sf14-encoded tRNAs, and not simply because the tRNA pool is larger.

#### tAI comparison of phage tRNA pool to combined tRNA pool

Finally, to explore the hypothesis of the all-destructive infection phenotype, we assumed that Sf14 degrades its host tRNAs and late infection proceeds with only phage tRNAs. In this case, we would expect at least the phage late genes to have the highest tAI values when calculated using the phage-only tRNA pool. Although one would assume the largest increase would be for Sf14, we also calculated tAI values for the host genome and gene groups.

As expected for the host, the average tAI for the *S. flexneri* 2457 T genome when using the phage tRNA pool was the lowest of all tRNA pools at 0.247, compared to an average tAI of 0.313 for the combined tRNA pool (p-value 2.20E-16) or 0.309 for the host-only pool. The average tAI when using phage tRNAs compared to the combined tRNA pool was significantly lower for all gene groups as well, with metabolism genes having tAI values of 0.225 vs. 0.330 (p-value 2.20E-16), biosynthesis genes values of 0.178 vs. 0.289 (p-value 01.05E-3), gene expression genes of 0.156 vs. 0.253 (p-value 9.16E-8), stress response and drug resistance genes of 0.117 vs. 0.217 (p-value 1.31E-4), and transport and miscellaneous genes of 0.208 vs. 0.305 (p-value 1.19E-5). These results are also shown in Table 7; Fig. 8B-C.

Conversely, the opposite trend was seen in Sf14, but this was not consistent or significant throughout the genome. For the Sf14 genome overall, the values when using the phage-only tRNA pool were significantly higher than those from the combined tRNA pool (0.281 vs. 0.230, p-value 7.32E-3). The early and middle genes showed a non-significant preference for the combined tRNA pool, but average tAI values for late genes were significantly higher when calculated using the phage-only tRNA pool versus the combined pool (0.379 vs. 0.304, p-value 0.035). This could be consistent with phage tRNA production during the later stages of infection if assuming an all-destructive infection phenotype. The hypothetical genes also showed a preference for the phage tRNA pool, but this difference was not significant. These data are shown in Fig. 8B, which depicts the average tAI values for both host and phage genomes and all gene groups when using the phage tRNA pool. A comparison of average tAI across genomes and gene groups when using the combined tRNA pool can be found in Fig. 8C. A comparison for all tRNA pools is shown in Fig. 8D.

### Mutations in Sf14-encoded tRNAs

Finally, we examined mutation patterns in the anticodon loops of Sf14-encoded tRNAs compared to the host-encoded tRNAs. This was done to test the hypothesis that phage-encoded tRNAs possess anticodon loop mutations that make them insensitive to host anticodon nucleases. We hypothesized that inconsistencies between the tRNAscan-SE 2.0 predicted model and the actual anticodon were related to mutations, but this was not the case. The structure of all Sf14 tRNAs and the corresponding *S. flexneri* 2457 T tRNAs were modeled using RNAfold [43] and compared. Because the Sf14 tRNA for Lys-CTT does not have a corresponding tRNA in the host, it was not included in the mutation analysis. Differences were found in the anticodon loops of 12 Sf14-encoded tRNAs compared to their corresponding host tRNAs, but these mutations occurred in both consistent and inconsistent tRNAs. This suggests the level of consistency is not explained by anticodon loop mutations. A summary of these mutations is presented in Table 8.

**Table 8:** Mutation patterns of Sf14 tRNAs, with changes at the specific position (pos) of the anticodon loop

					Anticodon Loop				
Pair_ID	Isotype		Anticodon		Sf14	2457T	Mutation	Consistent	
1	Pro		TGG		TTTGGGG	TTTGGGA	Pos 7: A→G	Yes	
2	Glu		TTC		CTTTCAA	CTTTCAC	Pos 7: C→A	No	
3	Met		CAT		CTCATTA	CTCATAA	Pos 6: A→T	No	
4	Asn		GTT		CTGTTAA	CTGTTAA	-	No	
5	Tyr		GTA		CTGTAAA	CTGTAAA	-	Yes	
6	Asp		GTC		CTGTCAC	CTGTCAC	-	Yes	
7	Lys		TTT		CTTTTAA	CTTTTAA	-	No	
8	Met		CAT		TTCATAA	CTCATAA	Pos 1: C→T	No	
9	Ile		GAT		CTGATAA	CTGATAA	-	No	
10	Arg		TCT		CTTCTAA	CTTCTAA	-	Yes	
11	Ser		TGA		CTTGAAA	CTTGAAA	-	Yes	
12	Leu		TAG		CTTAGAA	TTTAGGT	Pos 1: T→C; Pos 6: G→A; Pos 7: T→A	Yes	
13	Ala		TGC		TTTGCAA	TTTGCAC	Pos 7: C→A	Yes	
14	Gly		TCC		CTTCCAA	CTTCCAA	-	Yes	
15	Thr		TGT		CTTGTAA	CTTGTAA	-	No	
16	Val		TAC		CTTACAA	CTTACAA	-	No	
17	Leu		CAA		CTCAAAC	TTCAAAA	Pos 1: T→C; Pos 7: T→C	Yes	
18	Arg		ACG		CTACGAA	CTACGAA	-	Yes	
19	Gln		TTG		CTTTGAA	TTTTGAT	Pos 1: T→C; Pos 7: T→A	No	
20	Leu		TAA		TTTAAAA	TTTAAAA	-	Yes	
21	Gln		CTG		CTCTGAA	TTCTGAT	Pos 1: T→C; Pos 7: T→A	No	
22	His		GTG		CTGTGAA	TTGTGAT	Pos 1: T→C; Pos 7: T→A	No	
23	Phe		GAA		CTGAAAA	TTGAAAA	Pos 1: T→C	No	
24	Ser		GCT		TTGCTAA	CTGCTAA	Pos 1: C→T	No	
25	Cys		GCA		CTGCAAA	TTGCAAA	Pos 1: T→C	Yes	

If mutations were present, they most often occurred either two bases upstream (position 1) or two bases downstream (position 7) of the anticodons except tRNA-Leu-TAG, which had three mutations total at positions 1, 6, and 7; and one of the tRNA-Met, which had one mutation at position 6. Mutations in the anticodon loop are associated with anticodon nuclease resistance and changes in tRNA stability [20, 49, 50]. The host bacterium *S. flexneri* currently is known to carry only one anticodon nuclease, VapC, which targets the initiator tRNA-fMet [28]. Although Sf14 does encode two tRNAs for methionine, neither of these are classified as tRNA-fMet. Based on this, it is unlikely the mutations in the Sf14 tRNAs contribute to anticodon nuclease resistance.

**Fig. 9:**
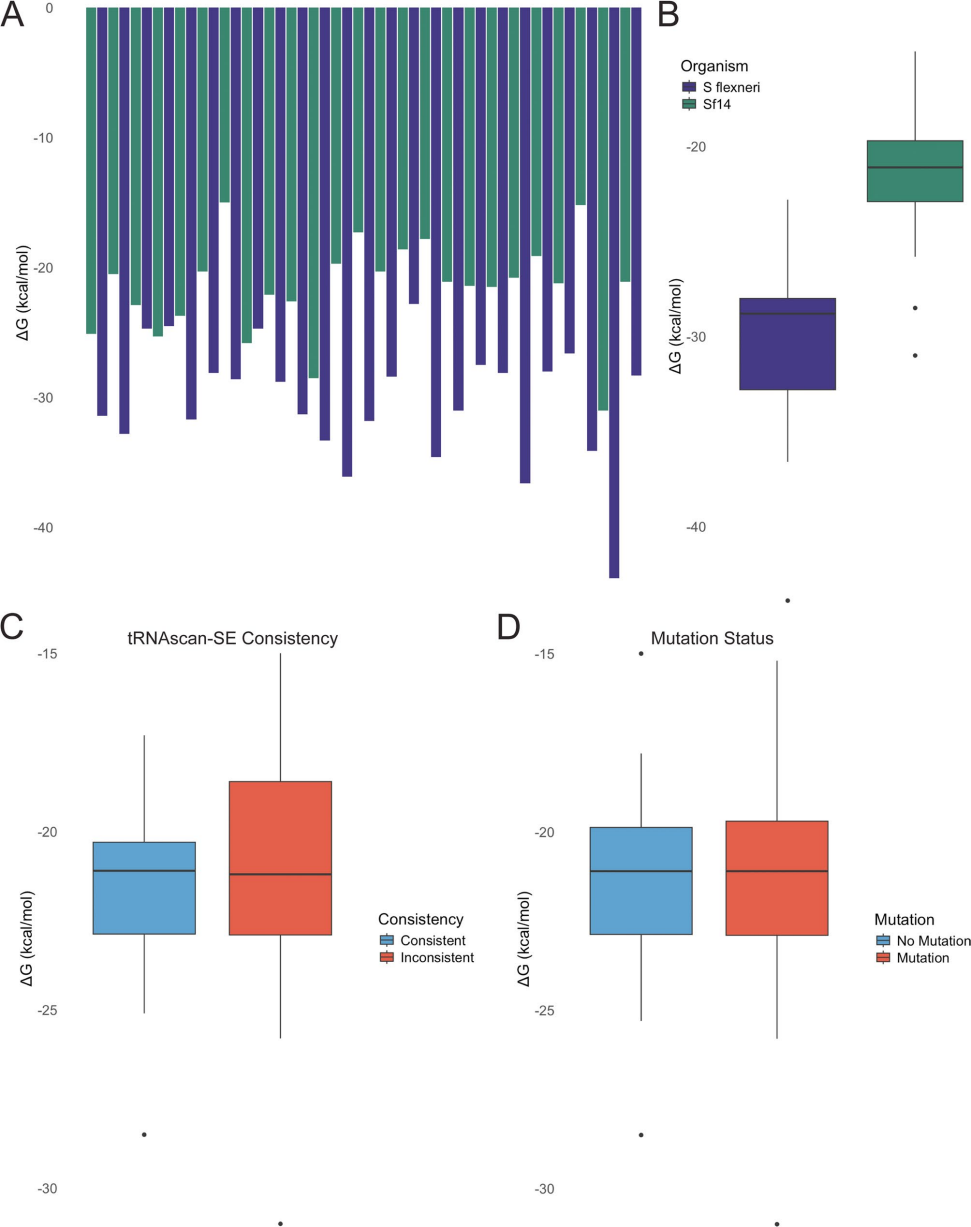
∆G Values of host and phage tRNAs. **A** Stacked bar graph of free energy values (∆G) for Sf14-encoded tRNAs and their *S. flexneri* 2457 T counterparts. **B** Distribution of ∆G values for Sf14-encoded tRNAs and their 2457 T counterparts. **C** Distribution of ∆G values for Sf14-encoded tRNAs by mutation status. **D** Distribution of ∆G values for Sf14-encoded tRNAs based on whether a tRNA was consistent or inconsistent with the tRNAscan-SE 2.0 predicted model

During mutation analysis, the free energy (∆G) for each tRNA in Sf14 and its corresponding host tRNA were calculated to examine the stability of each tRNA. It was discovered that overall, Sf14-encoded tRNAs are significantly less stable than the host tRNAs, with mean ∆G of −21.516 and − 30.308, respectively (p-value 2.27E-9). The ∆G values of the phage and host tRNAs are presented in Fig. 9A-B. We then asked whether tRNA stability was related to the presence or absence of mutations. To assess this, the ∆G values of phage tRNAs with mutations were compared to phage tRNAs without mutations. This revealed no significant correlation between mutation status and tRNA stability (p-value 0.850). Finally, whether tRNA stability was related to its consistency with the tRNAscan-SE 2.0 predicted model was then investigated, revealing no significant relationship (p-value 0.693). These two comparisons can be found in Fig. 9C-D. We also investigated the relationship between RSCU and ∆G for host and phage tRNAs, but found no correlation (data not shown). Based on these data, it is unlikely that mutations resulting in anticodon nuclease insensitivity drive the acquisition of tRNAs in phage Sf14. All data relating to these analyses is available in Supplementary Dataset 9.

## Discussion

It is known that phages rely on the host to translate their proteins, which may affect how their genes are encoded. If having similar codon biases with their hosts provides a selective advantage, this could ultimately result in having closely matched codon usage and GC content to that of their hosts [17, 48]. This is not the case for all phages though, and many phages encode their own tRNA genes [11, 12]. Although the phenomenon of encoding their own tRNAs has been known since the early days of phage biology, there is no single hypothesis that explains their presence in phage genomes. One of the leading hypotheses – the codon usage bias hypothesis – posits that phage-encoded tRNAs serve to compensate for any GC content and codon usage differences between the phage and its host. One example is *Escherichia* phage T4, which encodes an additional 8 tRNA genes to increase the translation efficiency of genes with a different codon bias from that of the host [8–10]. This could also provide a benefit during the initial stages of host range expansion, where the phage genome may not be as well-aligned with the new host codon usage or GC content [17, 18].

Another hypothesis suggests that phage-encoded tRNAs are required for later stages of infection when the phage has an all-destructive infection phenotype, providing tRNAs to continue translation in the absence of host-encoded tRNAs. Yang et al. (2021) demonstrated this with Vibriophage 2.275.O, which shows preference for the host tRNA pool early in infection but switches to preferring its own tRNA pool during later stages of infection [19]. The authors hypothesize this Vibriophage degrades the entirety of the host genome, including genes encoding host tRNAs. To continue translating viral proteins during later stages of infection, the phage must rely solely on its own tRNAs to translate viral proteins. Yet another hypothesis suggests that phage-encoded tRNAs may have anticodon loop mutations that make them insensitive to cleavage by the host tRNA anticodon nucleases that are produced as a response to viral infection. This phenomenon has been shown in the cluster C Mycobacteriophages [20].

Initially, we suspected that codon bias alone drove the acquisition of tRNA genes by the *Shigella* phage Sf14, as the GC content of the Sf14 genome – as well as the individual gene groups of early, middle, late, and hypothetical – have significantly lower GC content than both the *Shigella flexneri* 2457 T genome overall and the genes of interest examined here. Additionally, calculations of relative synonymous codon usage (RSCU) determined significant differences in codon usage for all but 8 codons between the host and phage genomes, as well as between host genes of interest and the different phage groups. However, the late genes encoded by Sf14 also have significantly different codon usage compared to the Sf14 early, middle, and hypothetical genes, indicating that genome-wide codon usage differences may not fully explain the presence of 26 phage-encoded tRNAs in the Sf14 genome.

Because the RSCU values of the Sf14 late genes compared to other groups of Sf14 genes are significantly different, the effect of these differences on translational efficiency was of interest. To examine this, tRNA adaptation index (tAI) was analyzed for both genomes and for specific gene groups [25–27]. At the genome level, differences in tAI between the host and phage were examined using their respective tRNA pools, revealing that Sf14 tAI values were significantly lower than those of the host. This suggests overall lower translational efficiency when Sf14 uses the tRNAs it encodes, in which case these tRNAs do not provide a genome-wide benefit on their own. We anticipated that many gene groups would follow a similar trend, with host gene groups having higher tAI than the phage groups. At the group level, however, we only saw significant differences only between the host metabolism genes and the phage hypothetical genes when each organism was using their respective tRNAs. For the early, middle, and late genes of Sf14, translational efficiency was roughly similar to the translational efficiency of the host gene groups. There were also significant differences in the tAI of Sf14 groups of middle and late genes compared to hypothetical genes. Unfortunately, since these genes are still hypothetical, the reasons for these differences remain elusive.

Next, the impact of the tRNA pool on the tAI of both host and phage genomes and their respective gene groups was examined. Three separate pools were independently used to calculate and analyze tAI, then compared: the host tRNA pool only, phage tRNA pool only, or a combined tRNA pool. Theoretically, having the larger tRNA pool of the host-only or the combination of host and phage tRNAs would increase efficiency of translation. This was reflected in the tAI values of both the *S. flexneri* 2457 T genome overall and the specific gene groups. With the exception of host biosynthesis genes, the tAI of the host genome and gene groups were – expectedly – significantly higher when using the host tRNA pool, which contains 100 tRNA genes, as compared to the phage tRNA pool, which contains only 26. Likewise, the *S. flexneri* 2457 T genome and gene groups showed significantly higher tAI values when using a combined tRNA pool compared to the host tRNA pool only, with the exception again of genes involved in biosynthesis and those involved in transport/miscellaneous activities. In following this pattern, the *S. flexneri* 2457 T genome and all gene groups had significantly higher tAI values when using the largest pool of combined tRNAs compared to using the smallest pool consisting of only phage tRNAs.

If the phage tRNAs only compensate for codon usage differences, it would be expected that their supplementation in the combined tRNA pool would have the highest tAI values like it did for the *Shigella* genome and gene groups. Although tAI values were indeed higher for Sf14 when calculated using the combined tRNA pool compared to the host-only tRNA pool, tAI was even higher for the Sf14 genome and late genes when using the phage tRNA pool only. Since the late genes especially seem to rely heavily on the phage tRNA pool for translational efficiency, this suggests the Sf14-encoded tRNAs are needed specifically during later stages of infection. Combined with the RSCU data, which showed codon usage differences for all gene groups, this suggests Sf14 may have an all-destructive infection phenotype.

One specific codon was examined in depth. The AAG codon has a complementary tRNA in the Sf14 genome, but not in the host *S. flexneri* 2457 T genome. How this codon affects codon usage bias and translational efficiency were therefore of interest. In examining the RSCU of lysine, with only codons AAG or AAA, 40 out of 131 (30.5%) Sf14-encoded genes have an RSCU ≥ 1 for codon AAG, and 26 (19.8%) have an RSCU > 1 for the codon AAG. This is dramatically higher than the *S. flexneri* 2457 T RSCU for the AAG lysine codon. Of 4,495 coding sequences, only 406 (9.0%) have an RSCU for AAG ≥ 1, with only 252 (5.6%) genes having an RSCU > 1 for this codon. These data indicate that Sf14 does utilize the AAG codon more frequently than the host, which may explain the presence of tRNA-Lys-CTT in the phage genome. Since 2457 T does not encode this tRNA but still uses the codon preferentially for some genes, it likely uses wobble base-pairing during translation of these gene products. It has been established that changes to wobble base pairing can affect the virulence of *S. flexneri* by altering the translation of virulence factor VirF [51–53]. Wobble base pairing of the tRNA-Lys-UUU to AAG codons likely also occurs for a subset of genes, which may be affected by environmental factors similar to VirF. Of the 252 genes that use AAG more frequently, 55 are pseudogenes and another 45 are likely from mobile genetic elements, including prophages that are likely no longer functional (see Supplementary Table 5, 2457T_high_AAG). The remaining genes appear to be involved in a variety of functions, including metabolism, nutrient transport, gene expression, translation, and cell division. Whether these genes are significantly impacted during infection and/or whether they themselves affect the outcome of infection has yet to be determined.

Lastly, the possibility of anticodon loop mutations contributing to tRNA acquisition for evasion of host anticodon nucleases was investigated [20, 21]. While multiple tRNAs were found to contain anticodon loop mutations, the nuclease evasion hypothesis is unlikely. The host *S. flexneri* 2457 T is only known to carry one anticodon nuclease, VapC. This nuclease targets the tRNA-fMet [28], which is not present in the Sf14 genome. Finally, as part of our mutation analysis, free energy values (∆G) for host and phage tRNAs were calculated and compared. The results showed phage tRNAs had significantly less stable tRNAs than the corresponding host tRNAs, but this decrease in stability was related to neither the anticodon loop mutations nor the tRNA’s consistency with tRNAscan-SE 2.0 predicted models. Thus, anticodon loop mutations are unlikely to be a driving force behind tRNA acquisition in *Shigella* phage Sf14.

## Conclusions

From this work, a combination of codon usage bias differences and increased translational efficiency of certain genes are likely the driving forces behind the acquisition of tRNA genes by *Shigella* phage Sf14. This is supported by several pieces of evidence namely: (1) the significant differences in RSCU for all Sf14 gene groups compared to the *S. flexneri* 2457 T gene groups, and (2) the significantly higher tAI values for the Sf14 genome, late genes, and hypothetical genes when relying on the phage tRNA pool only for translation. Since the phage-encoded tRNAs are utilized more heavily later in infection, we hypothesize Sf14 possesses an all-destructive phenotype, where the host genome—including genes encoding tRNAs—is ultimately degraded and structural genes rely more heavily on phage-encoded tRNAs for the translation of late genes. While significant differences in codon usage bias do exist between Sf14 and the host gene groups, the phage-encoded tRNAs appear to specifically increase the translational efficiency of late-encoded genes. We cannot entirely rule out that this is an important role of these tRNAs. Follow-up studies examining gene expression and tRNA abundance in both the host and phage are required to investigate these hypotheses and to differentiate between total tRNA replacement, as in the all-destructive infection phenotype, or tRNA supplementation to facilitate translation of late genes.

## Supplementary Information

Supplementary Material 1. Supplementary figure S1: median RSCU values of the Sf14 and S. flexneri 2457 T genomes Stacked bar graph comparing the median RSCU values of each codon in Sf14 and host *S. flexneri* 2457T. Asterisks indicate the difference in RSCU is significant. Supplementary figure S2: average RSCU values for the S. flexneri 2457 T genome and all gene groups of interest Bar graphs showing the average RSCU values for all codons in 2457 T for (A) genome, (B) metabolism genes, (C) biosynthesis genes, (D) gene expression genes, (E) stress response and drug resistance genes, and (F) transport and miscellaneous genes. Supplementary figure S3: average RSCU values for Sf14 genome and all Sf14 gene groups Bar graphs showing the average RSCU values for all codons in Sf14 (A) genome, (B) early genes, (C) middle genes, (D) late genes, and (E) hypothetical genes. Supplementary figure S4: PCA plot of RSCU scores for all codons in the Sf14 and S. flexneri 2457 T gene groups Genes are assigned a color based on their group, as indicated in the key on the right. An unlabeled version of this plot is available in the text (Fig. 4). Some labels are hidden due to multiple overlapping observations. Supplementary figure S5: tAI values for both Sf14 and S. flexneri 2457 T genomes and host genes of interest Bar graphs depicting the tAI values for the (A) host genome, (B) host genes of interest, (C) Sf14 genome; (D) Boxplot of the distribution of tAI values in *S. flexneri* 2457 T and Sf14. Supplementary figure S6: Sf14-encoded tRNA mutation frequency by isotype and position (A) Bar graph of the mutation frequency for Sf14 tRNAs according to their isotype. (B) Bar graph of the frequency of mutations at each anticodon loop position. Supplementary figure S7: comparison of tRNA ∆G values between Sf14 and S. flexneri 2457 T Free energy (∆G) values for Sf14-encoded tRNAs and their counterparts in 2457T. Sf14 ∆G values are on the y-axis, and 2457 T ∆G values are on the x-axis. Supplementary figure S8: ∆∆G values (Sf14 – S. flexneri 2457 T) and the impact of mutation position on ∆∆G Dot plot of the mutation position (x-axis) of Sf14 mutated tRNAs as it relates to ∆∆G values (y-axis). Position “0” indicates no mutations were found. Supplementary figure S9: secondary structure of four representative mutated Sf14-encoded tRNAs Four Sf14-encoded tRNAs were modeled and compared to their host counterparts, with mutations in the anticodon loops highlighted in yellow. The tRNAs modeled were (A) tRNA-Leu-CAA, (B) tRNA-Leu-TAG, (C) tRNA-Phe-GAA, and (D) tRNA-Gln-TTG.

Supplementary Material 2. Supplementary dataset S1: GC content analysis and statistics Values for total, GC1, GC2, and GC3 for *S. flexneri* 2457 T and phage Sf14 genome and individual genes. Includes tabs for all Dunn’s tests and a summary of statistics. Supplementary dataset S2: RSCU analysis and statistics Relative Synonymous Codon Usage values for the *S. flexneri* 2457 T and phage Sf14 genome and genes. Specific genes of interest are grouped in individual tabs. Statistics are available as raw values, then as a summary in the final tab. Supplementary dataset S3: tRNAscan-SE 2.0 output Total tRNAs predicted for *S. flexneri* 2457 T and phage Sf14. Includes tRNA location in the genome, predicted isotype, isotype model, anticodon, and scores. Supplementary dataset S4: tAI analysis and statistics Values for tRNA Adaptation Index for all genes in the *S. flexneri* 2457 T and Sf14 genome, plus genes of interest. Statistics are available in the final tab. Supplementary dataset S5: Lysine-AAG and Lysine-AAA usage analysis Genes in both *S. flexneri* 2457 T and the Sf14 genome with tAI ≥ 0.5 and their usage of Lysine-AAG vs. Lysine-AAA tRNAs. Supplementary dataset S6: tAI analysis using the host-only tRNA pool Values for tRNA Adaptation Index for all genes in the *S. flexneri* 2457 T and Sf14 genome, plus genes of interest, when using *only* the host-encoded tRNAs. Statistics are available in the final tab. Supplementary dataset S7: tAI analysis using the phage-only tRNA pool Values for tRNA Adaptation Index for all genes in the *S. flexneri* 2457 T and Sf14 genome, plus genes of interest, when using *only* the phage-encoded tRNAs. Statistics are available in the final tab. Supplementary dataset S8: tAI analysis using the combined tRNA pool Values for tRNA Adaptation Index for all genes in the *S. flexneri* 2457 T and Sf14 genome, plus genes of interest, when using *both* the host- and phage-encoded tRNAs. Statistics are available in the final tab. Supplementary dataset S9: comparison of anticodon loops between host- and phage-encoded tRNAs Includes the anticodon and anticodon loop sequences for all pairs of tRNAs, including differences between the two, ΔG and ΔΔG values, and statistics.

## Data Availability

All data generated or analyzed during this study are included in this article and its supplementary information files. Genomes of the organisms used in this study are in the National Center for Biotechnology Information (NCBI) GenBank database under accession numbers [NC_004741] (https://www.ncbi.nlm.nih.gov/nuccore/NC_004741.1) (Shigella flexneri 2457 T) and [NC_042075] (https://www.ncbi.nlm.nih.gov/nuccore/NC_042075.1) (Shigella phage Sf14).
