# Supplementary material for: A computational investigation into the role of tRNAs encoded by Shigella phage Sf14
Source: BMC Genomics. 2025 Sep 29;26:858. doi: 10.1186/s12864-025-11998-9 (PMC12482277; doi:10.1186/s12864-025-11998-9)

Supporting Information

Supplementary Figures

Supplementary Figures	Page
**Figure S1:** Median RSCU values of the Sf14 and *S. flexneri* 2457T genomes.	**2**
**Figure S2:** Average RSCU values for the *S. flexneri* 2457T genome and all gene groups of interest.	**3**
**Figure S3:** Average RSCU values for Sf14 genome and all Sf14 gene groups.	**6**
**Figure S4:** PCA plot of RSCU scores for all codons in the Sf14 and *S. flexneri* 2457T gene groups.	**9**
**Figure S5:** tAI values for both Sf14 and *S. flexneri* 2457T genomes and host genes of interest.	**10**
**Figure S6:** Sf14-encoded tRNA mutation frequency by isotype and position.	**12**
**Figure S7:** Comparison of tRNA ΔG values between Sf14 and *S. flexneri* 2457T.	**13**
**Figure S8:** ΔΔG values (Sf14 – *S. flexneri* 2457T) and the impact of mutation position on ΔΔG.	**14**
**Figure S9:** Secondary structure of four representative mutated Sf14-encoded tRNAs.	**15**

Supplementary Datasets

Dataset S1: GC content analysis and statistics. Values for total, GC1, GC2, and GC3 for *S. flexneri* 2457T and phage Sf14 genome and individual genes. Includes tabs for all Dunn's tests and a summary of statistics.

Dataset S2: RSCU analysis and statistics. Relative Synonymous Codon Usage values for the *S. flexneri* 2457T and phage Sf14 genome and genes. Specific genes of interest are grouped in individual tabs. Statistics are available as raw values, then as a summary in the final tab.

Dataset S3: tRNAscan-SE 2.0 output. Total tRNAs predicted for *S. flexneri* 2457T and phage Sf14. Includes tRNA location in the genome, predicted isotype, isotype model, anticodon, and scores.

Dataset S4: tAI analysis and statistics. Values for tRNA Adaptation Index for all genes in the *S. flexneri* 2457T and Sf14 genome, plus genes of interest. Statistics are available in the final tab.

Dataset S5: Lysine-AAG and Lysine-AAA usage analysis. Genes in both *S. flexneri* 2457T and the Sf14 genome with tAI ≥ 0.5 and their usage of Lysine-AAG vs. Lysine-AAA tRNAs.

Dataset S6: tAI analysis using the host-only tRNA pool. Values for tRNA Adaptation Index for all genes in the *S. flexneri* 2457T and Sf14 genome, plus genes of interest, when using *only* the host-encoded tRNAs. Statistics are available in the final tab.

Dataset S7: tAI analysis using the phage-only tRNA pool. Values for tRNA Adaptation Index for all genes in the *S. flexneri* 2457T and Sf14 genome, plus genes of interest, when using *only* the phage-encoded tRNAs. Statistics are available in the final tab.

Dataset S8: tAI analysis using the combined tRNA pool. Values for tRNA Adaptation Index for all genes in the *S. flexneri* 2457T and Sf14 genome, plus genes of interest, when using *both* the host- and phage-encoded tRNAs. Statistics are available in the final tab.

Dataset S9: Comparison of anticodon loops between host- and phage-encoded tRNAs. Includes the anticodon and anticodon loop sequences for all pairs of tRNAs, including differences between the two, ΔG and ΔΔG values, and statistics.

Supplementary Figure S1: Median RSCU values of the Sf14 and *S. flexneri* 2457T genomes. Stacked bar graph comparing the median RSCU values of each codon in Sf14 and host *S. flexneri* 2457T. Asterisks indicate the difference in RSCU is significant.

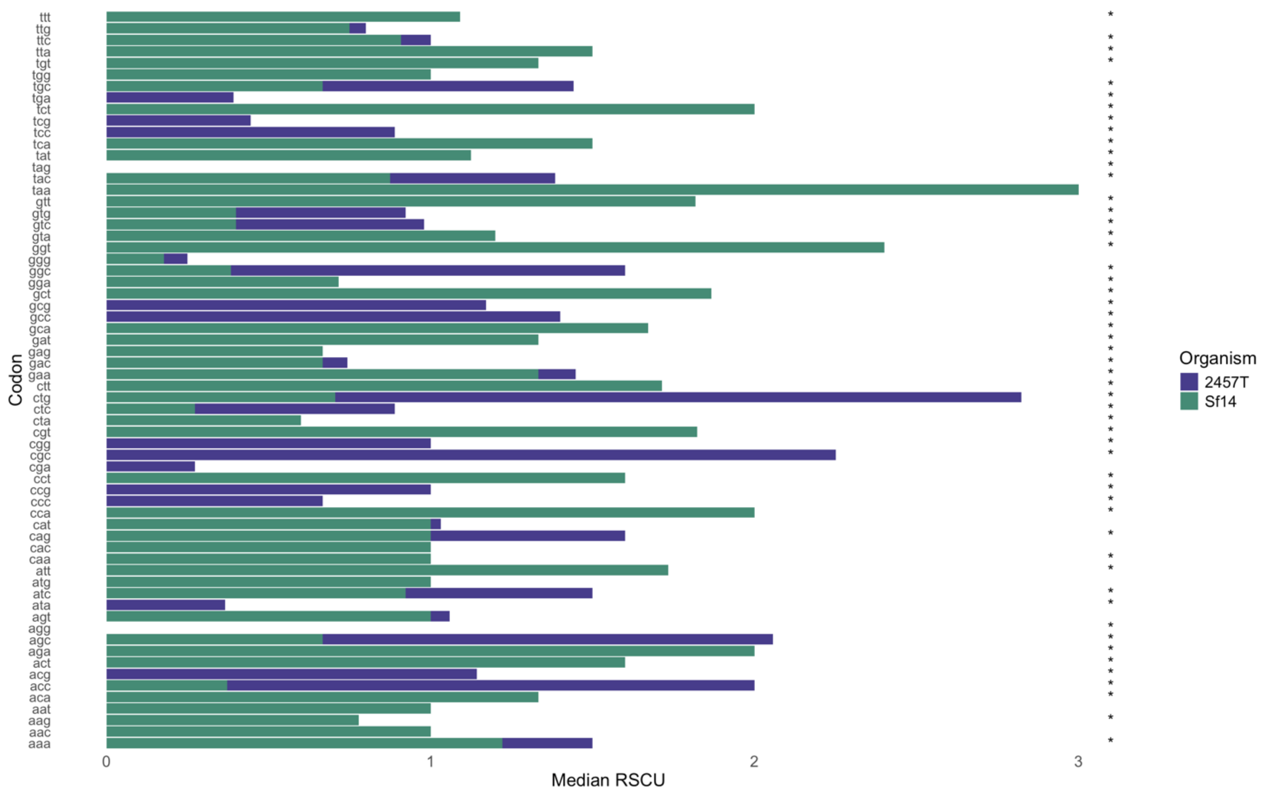

Supplementary Figure S2: Average RSCU values for the *S. flexneri* 2457T genome and all gene groups of interest. Bar graphs showing the average RSCU values for all codons in 2457T for A) genome, B) metabolism genes, C) biosynthesis genes, D) gene expression genes, E) stress response and drug resistance genes, and F) transport and miscellaneous genes.

A.

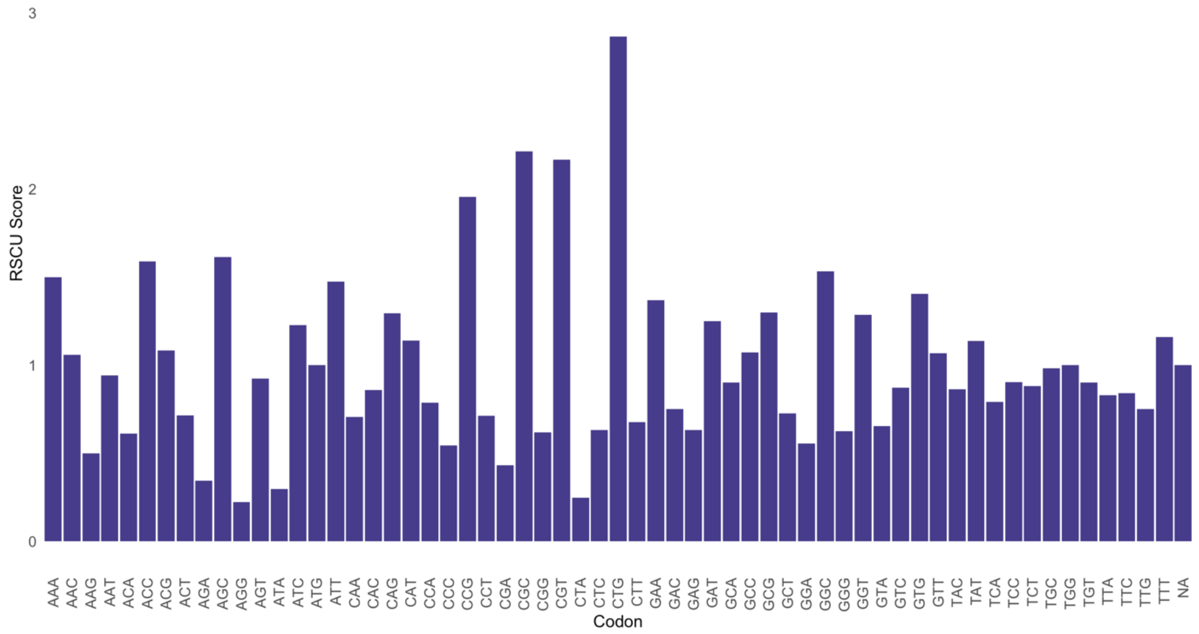

B.

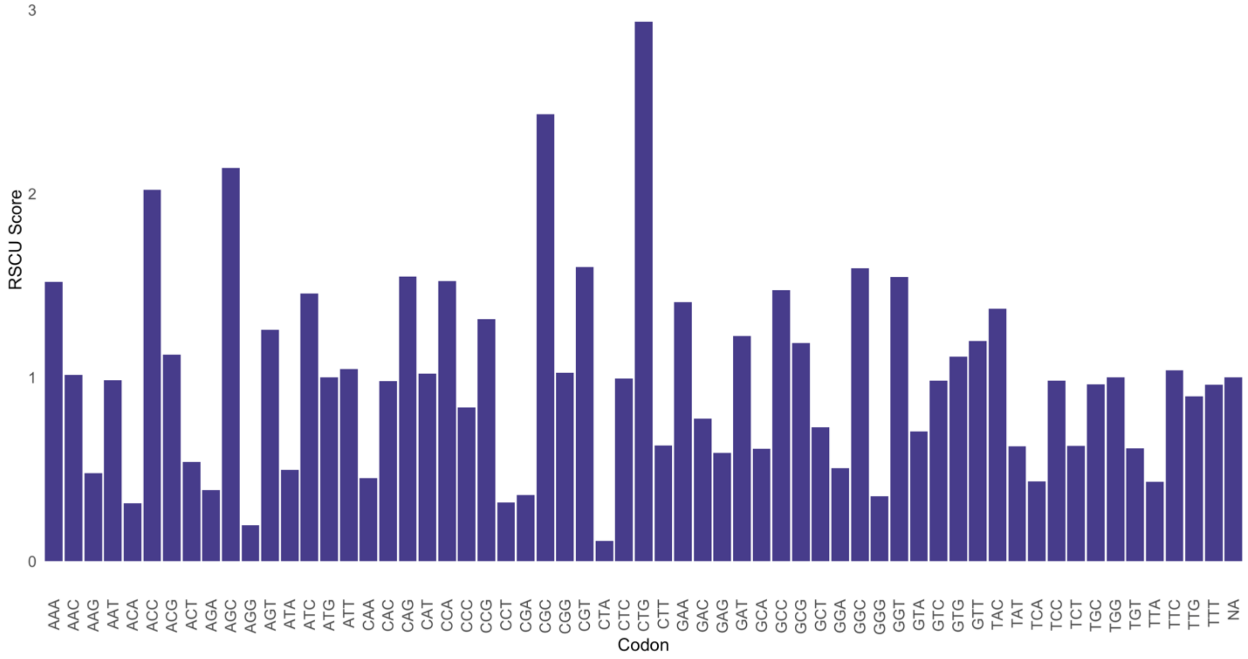

C.

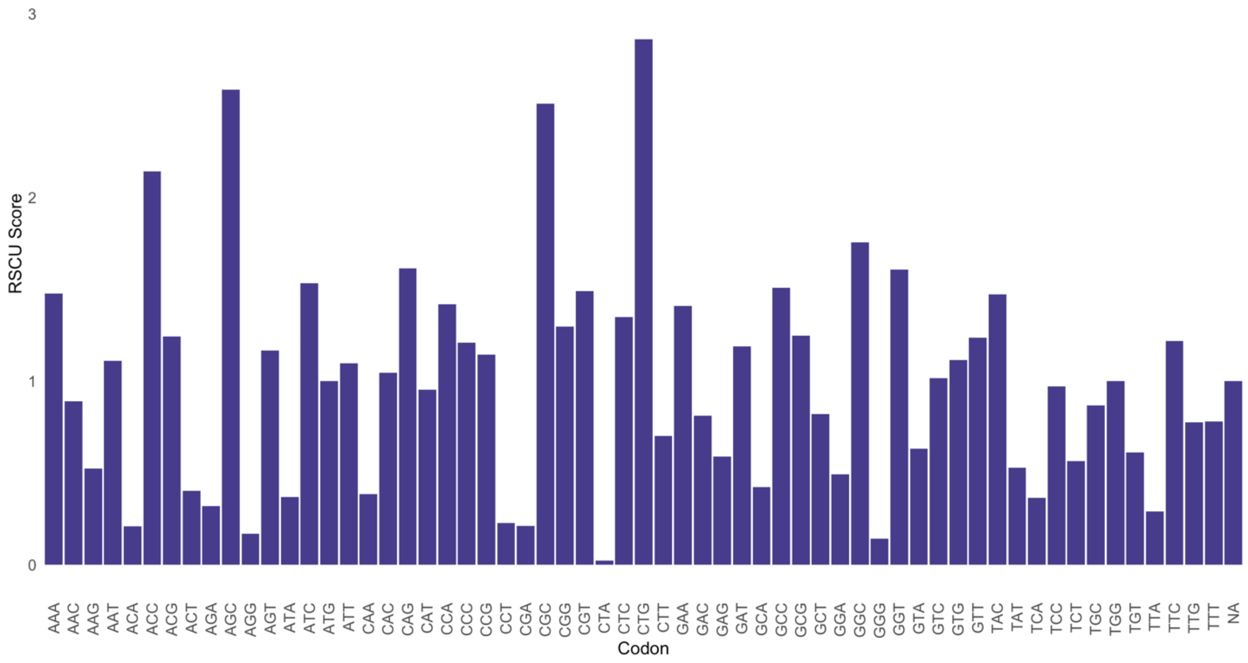
RSCU Score
0
1
2
3
Codon
AAA AAC AAG AAT ACA ACC ACG ACT AGA AGC AGG AGT ATA ATC ATG ATT CAA CAC CAG CAT CCA CCC CCG CCT CGA CGC CGG CGT CTA CTC CTG CTT GAA GAC GAG GAT GCA GCC GCG GCT GGA GGC GGG GGT GTA GTC GTG GTT TAC TAT TCA TCC TCT TGC TGG TGT TTA TTC TTG TTT NA

D.

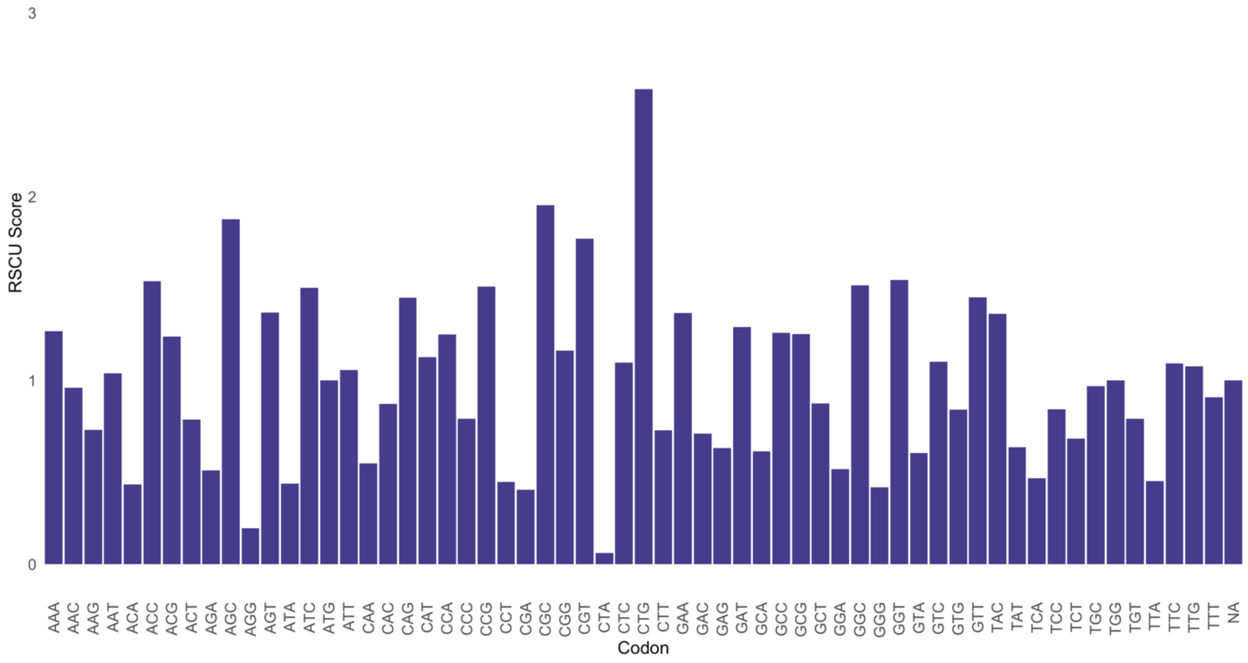
RSCU Score
0
1
2
3
Codon
AAA AAC AAG AAT ACA ACC ACG ACT AGA AGC AGG AGT ATA ATC ATG ATT CAA CAC CAG CAT CCA CCC CCG CCT CGA CGC CGG CGT CTA CTC CTG CTT GAA GAC GAG GAT GCA GCC GCG GCT GGA GGC GGG GGT GTA GTC GTG GTT TAC TAT TCA TCC TCT TGC TGG TGT TTA TTC TTG TTT NA

E.

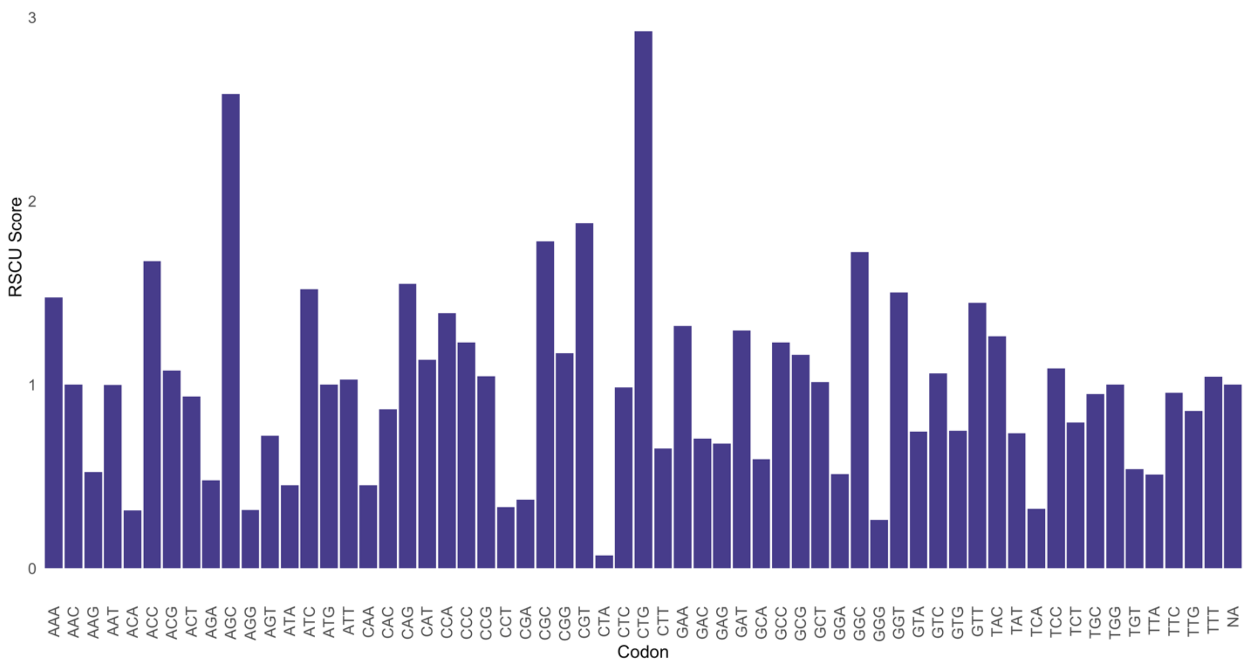
RSCU Score
Codon
0
1
2
3
AAA AAC AAG AAT ACA ACC ACG ACT AGA AGC AGG AGT ATA ATC ATG ATT CAA CAC CAG CAT CCA CCC CCG CCT CGA CGC CGG CGT CTA CTC CTG CTT GAA GAC GAG GAT GCA GCC GCG GCT GGA GGC GGG GGT GTA GTC GTG GTT TAC TAT TCA TCC TCT TGC TGG TGT TTA TTC TTG TTT NA

F.

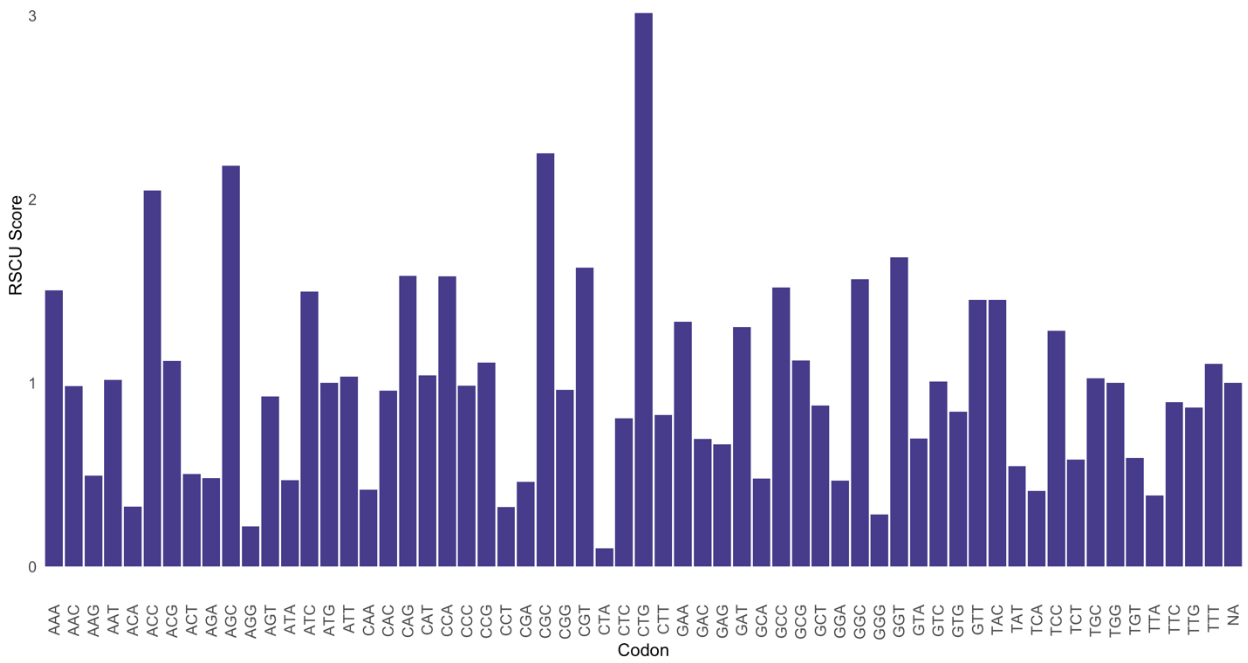
RSCU Score
Codon
0
1
2
3
AAA AAC AAG AAT ACA ACC ACG ACT AGA AGC AGG AGT ATA ATC ATG ATT CAA CAC CAG CAT CCA CCC CCG CCT CGA CGC CGG CGT CTA CTC CTG CTT GAA GAC GAG GAT GCA GCC GCG GCT GGA GGC GGG GGT GTA GTC GTG GTT TAC TAT TCA TCC TCT TGC TGG TGT TTA TTC TTG TTT NA

Supplementary Figure S3: Average RSCU values for Sf14 genome and all Sf14 gene groups. Bar graphs showing the average RSCU values for all codons in Sf14 A) genome, B) early genes, C) middle genes, D) late genes, and E) hypothetical genes.

A.

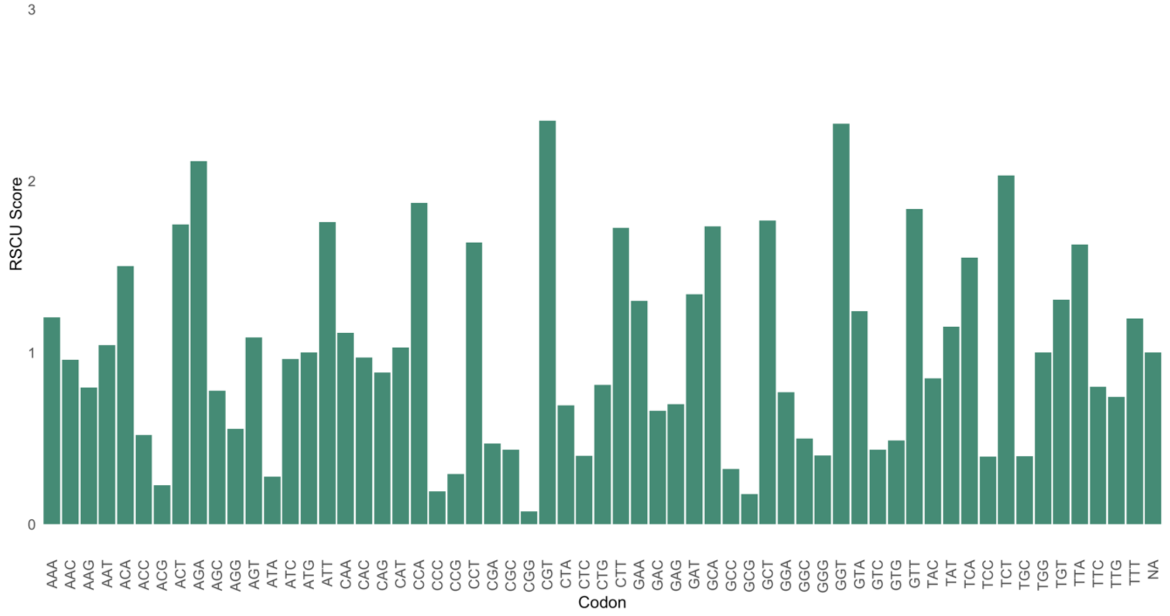

B.

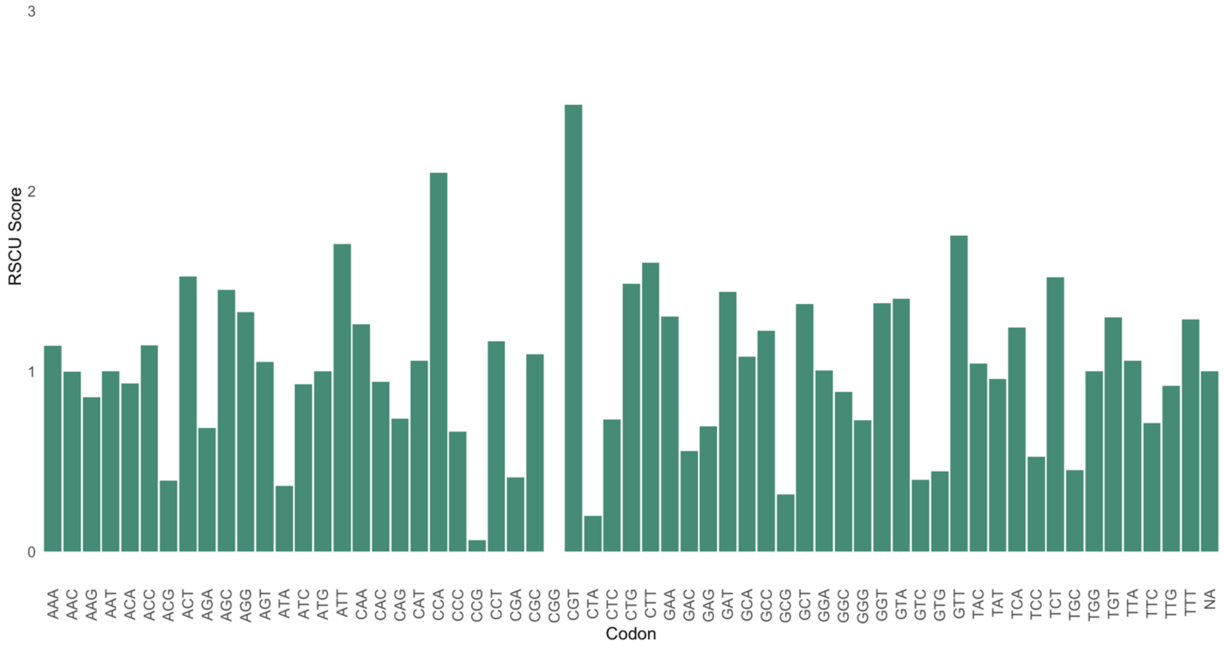

C.

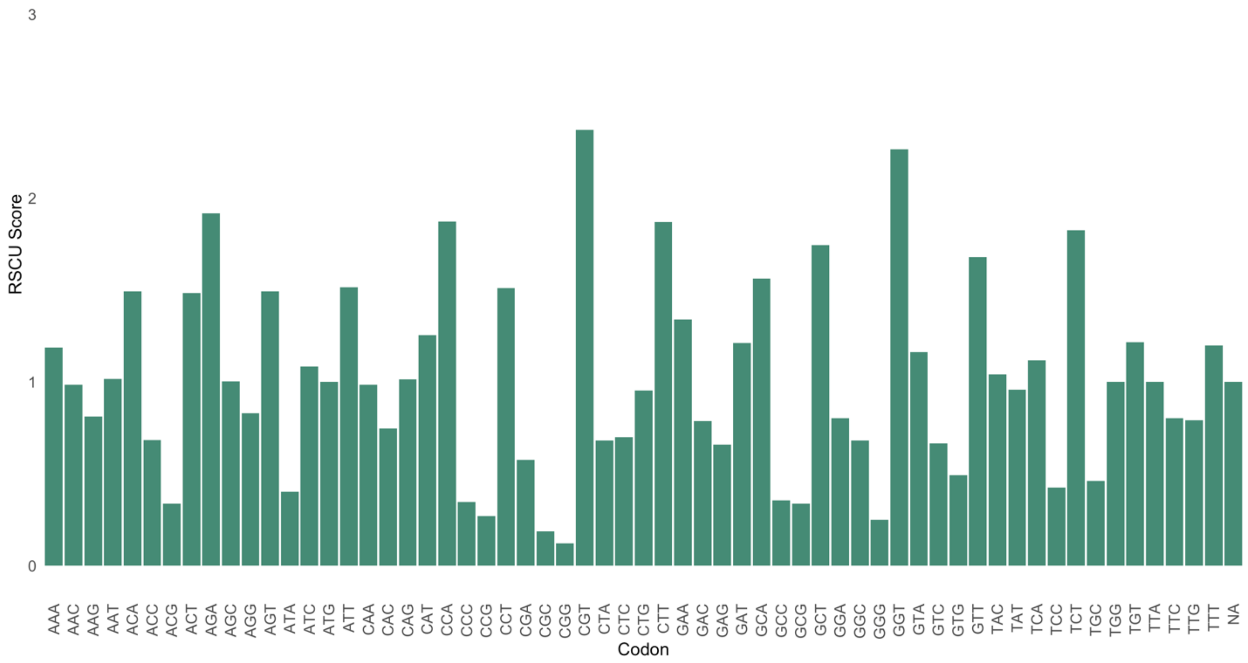
RSCU Score
Codon
0
1
2
3
AAA AAC AAG AAT ACA ACC ACG ACT AGA AGC AGG AGT ATA ATC ATG ATT CAA CAC CAG CAT CCA CCC CCG CCT CGA CGC CGG CGT CTA CTC CTG CTT GAA GAC GAG GAT GCA GCC GCG GCT GGA GGC GGG GGT GTA GTC GTG GTT TAC TAT TCA TCC TCT TGC TGG TGT TTA TTC TTG TTT NA

D.

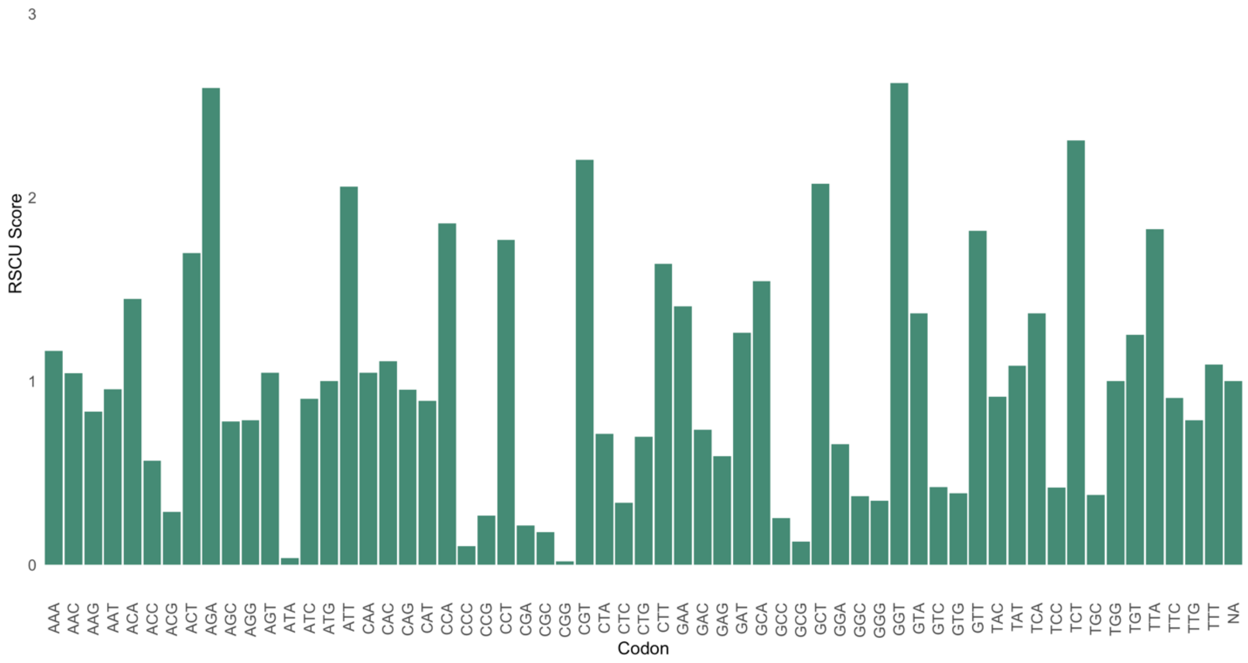
RSCU Score
Codon
0
1
2
3
AAA AAC AAG AAT ACA ACC ACG ACT AGA AGC AGG AGT ATA ATC ATG ATT CAA CAC CAG CAT CCA CCC CCG CCT CGA CGC CGG CGT CTA CTC CTG CTT GAA GAC GAG GAT GCA GCC GCG GCT GGA GGC GGG GGT GTA GTC GTG GTT TAC TAT TCA TCC TCT TGC TGG TGT TTA TTC TTG TTT NA

E.

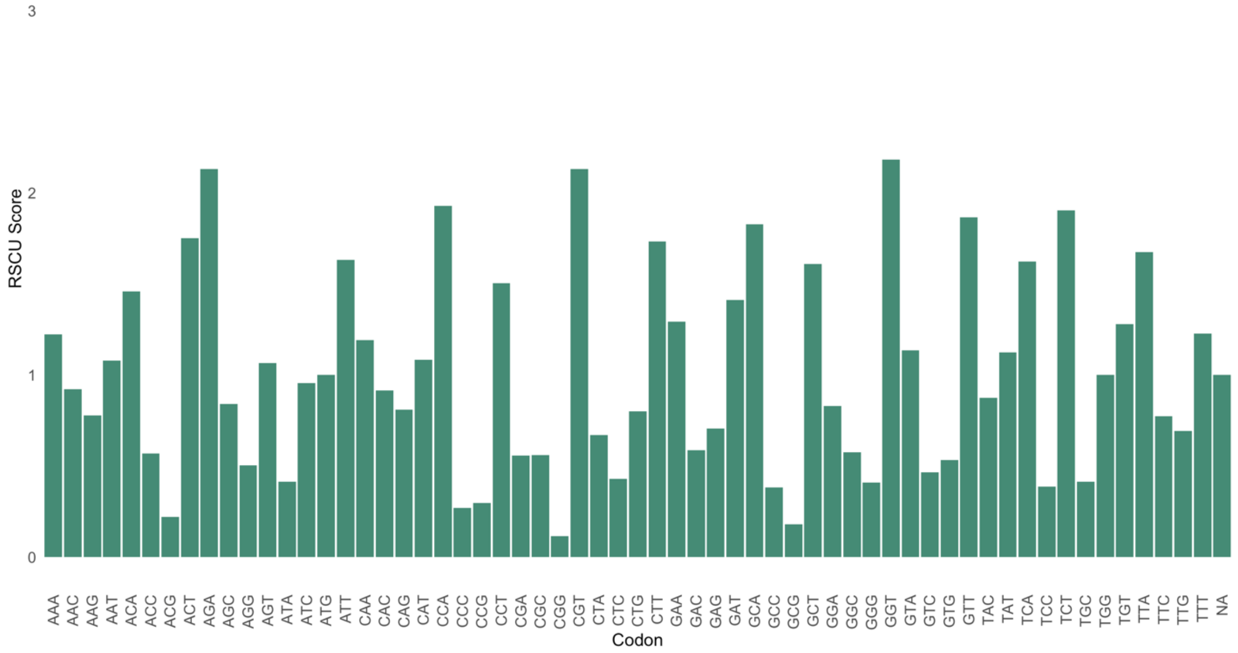

RSCU Score
Codon
0
1
2
3
AAA
AAC
AAG
AAT
ACA
ACC
ACG
ACT
AGA
AGC
AGG
AGT
ATA
ATC
ATG
ATT
CAA
CAC
CAG
CAT
CCA
CCC
CCG
CCT
CGA
CGC
CGG
CGT
CTA
CTC
CTG
CTT
GAA
GAC
GAG
GAT
GCA
GCC
GCG
GCT
GGA
GGC
GGG
GGT
GTA
GTC
GTG
GTT
TAC
TAT
TCA
TCC
TCT
TGC
TGG
TGT
TTA
TTC
TTG
TTT
NA

Supplementary Figure S4: PCA plot of RSCU scores for all codons in the Sf14 and *S. flexneri* 2457T gene groups. Genes are assigned a color based on their group, as indicated in the key on the right. An unlabeled version of this plot is available in the text (Figure 4). Some labels are hidden due to multiple overlapping observations.

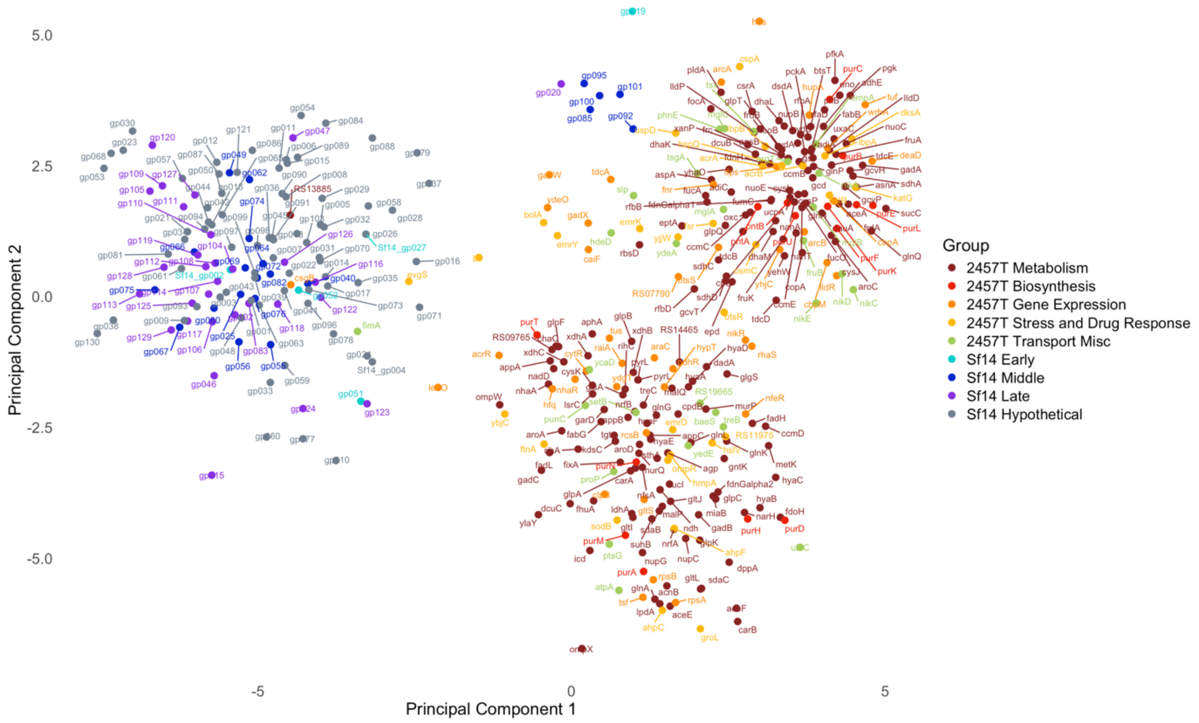

Supplementary Figure S5: tAI values for both Sf14 and *S. flexneri* 2457T genomes and host genes of interest. Bar graphs depicting the tAI values for the A) host genome, B) host genes of interest, C) Sf14 genome; D) Boxplot of the distribution of tAI values in *S. flexneri* 2457T and Sf14.

A.

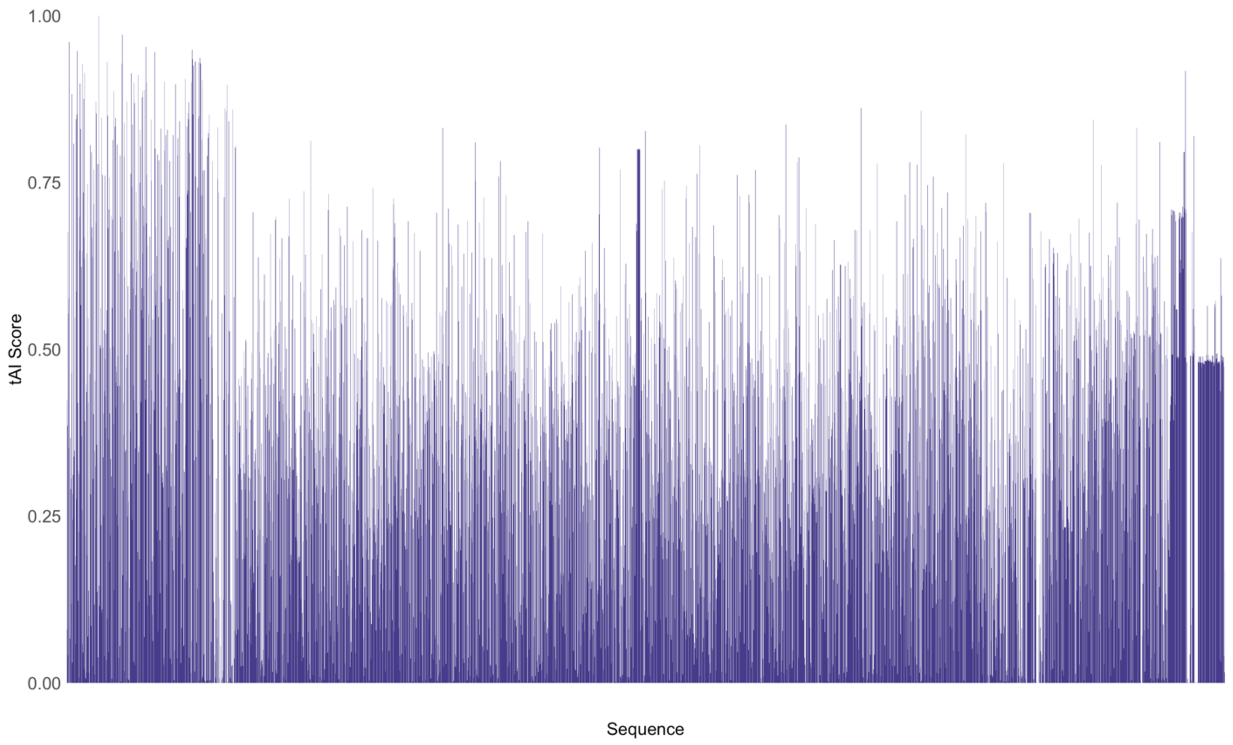

B.

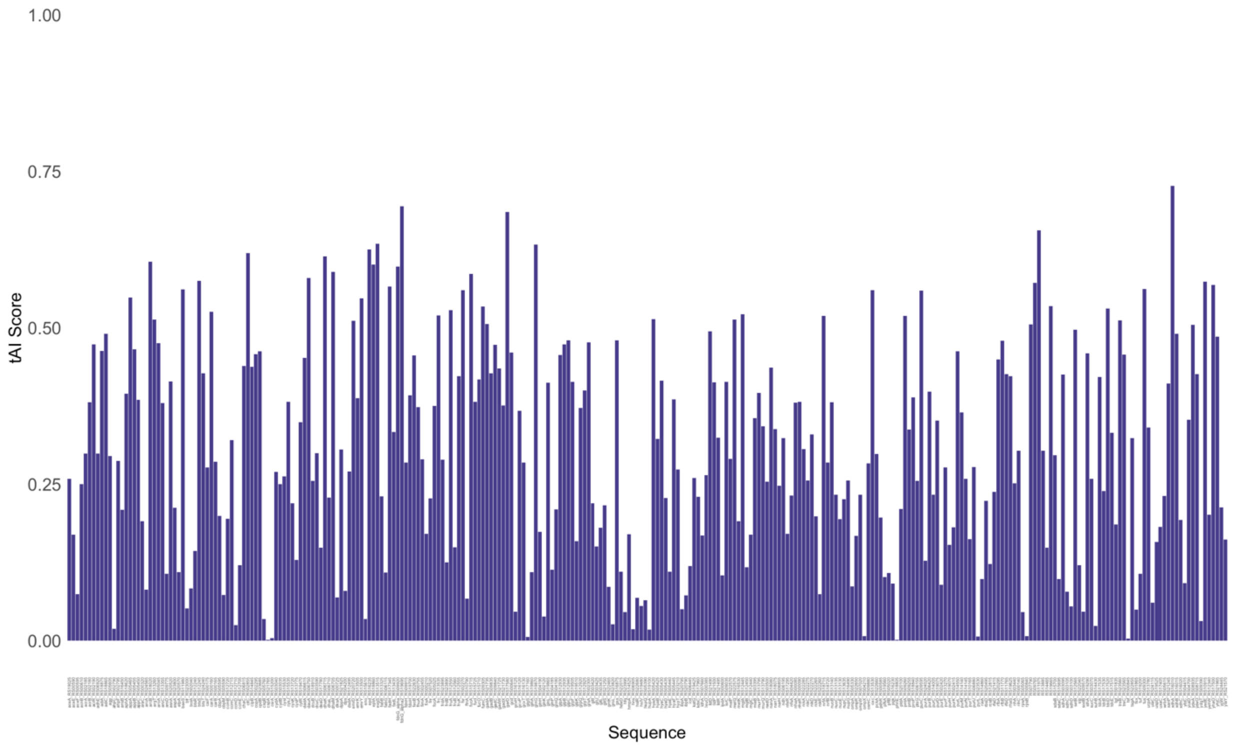

C.

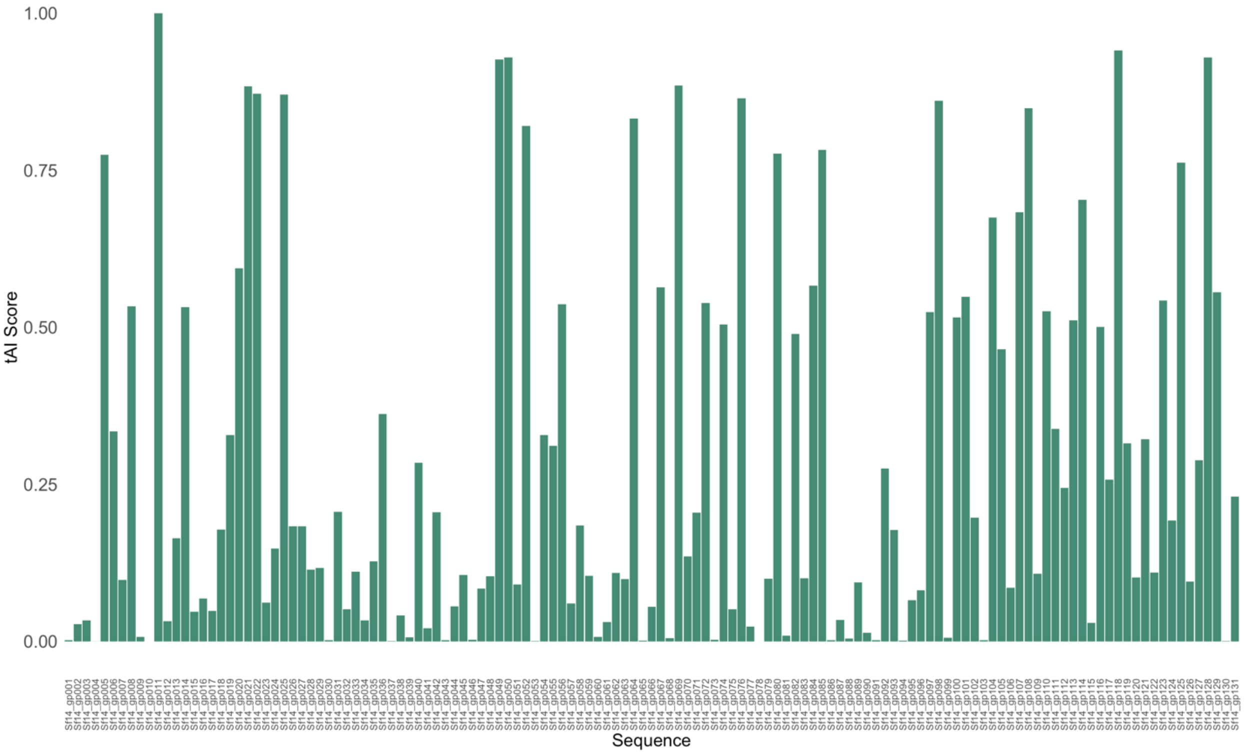

1.00
0.75
0.50
0.25
0.00
tAI Score
Sequence

D.

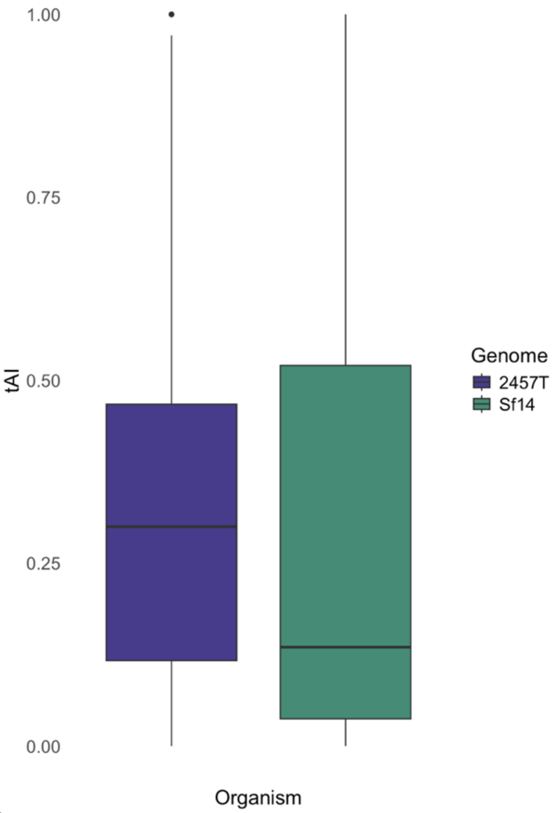

1.00
0.75
0.50
0.25
0.00
tAI
Organism
Genome
2457T
Sf14

Supplementary Figure S6: Sf14-encoded tRNA mutation frequency by isotype and position. A) Bar graph of the mutation frequency for Sf14 tRNAs according to their isotype. B) Bar graph of the frequency of mutations at each anticodon loop position.

A.

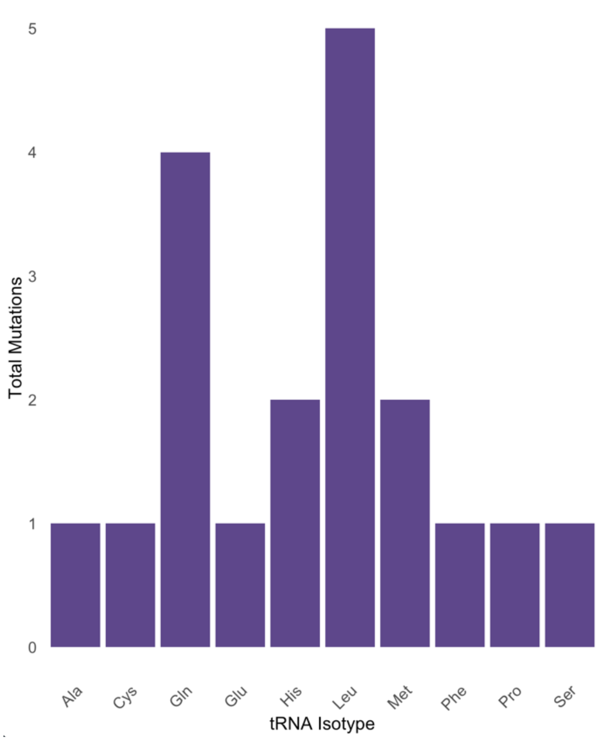

B.

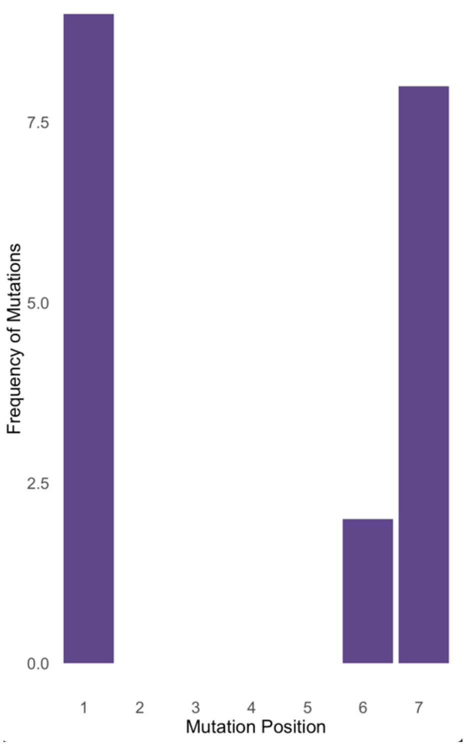

Supplementary Figure S7: Comparison of tRNA ΔG values between Sf14 and *S. flexneri* 2457T. Free energy (ΔG) values for Sf14-encoded tRNAs and their counterparts in 2457T. Sf14 ΔG values are on the y-axis, and 2457T ΔG values are on the x-axis.

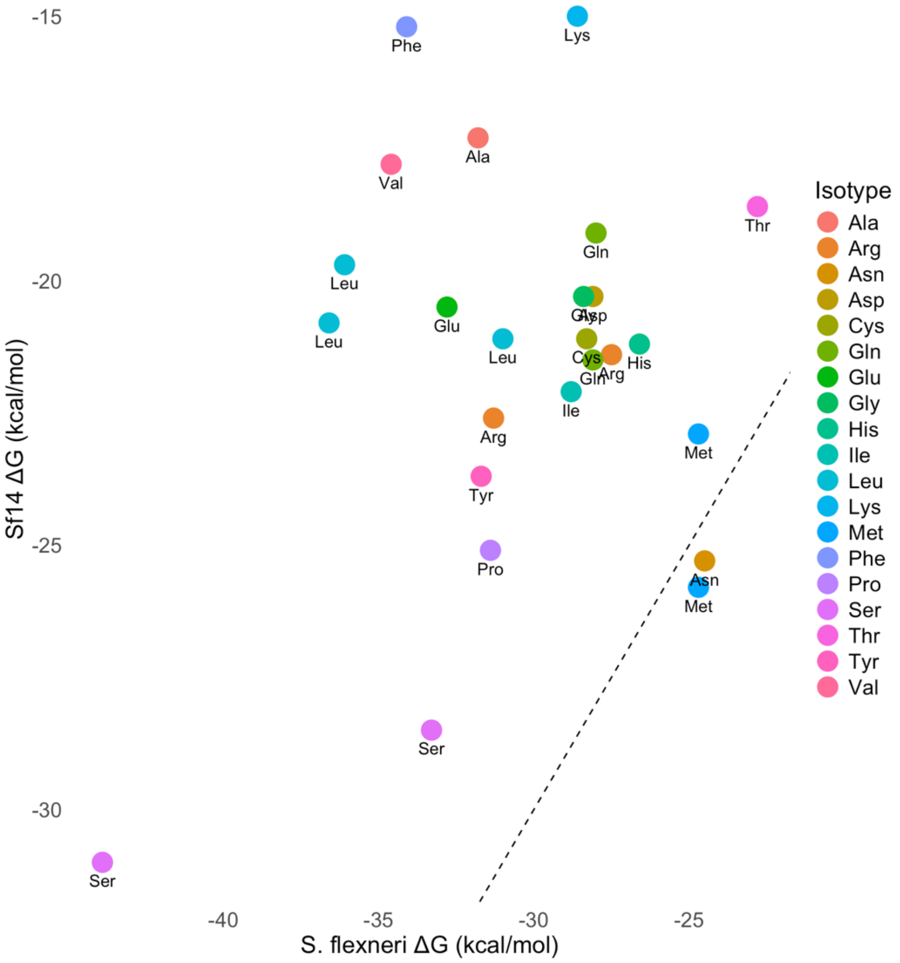

Supplementary Figure S8: ΔΔG values (Sf14 – *S. flexneri* 2457T) and the impact of mutation position on ΔΔG. Dot plot of the mutation position (x-axis) of Sf14 mutated tRNAs as it relates to ΔΔG values (y-axis). Position "0" indicates no mutations were found.

A.

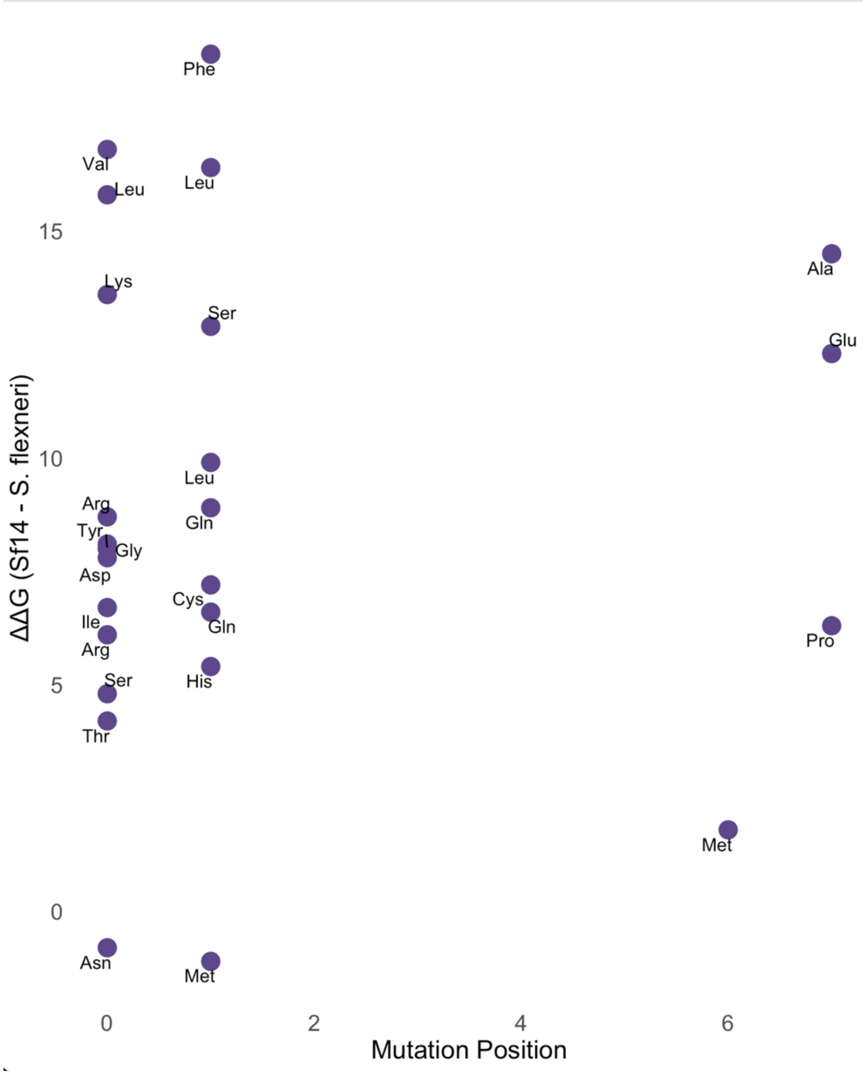

Supplementary Figure S9: Secondary structure of four representative mutated Sf14-encoded tRNAs. Four Sf14-encoded tRNAs were modeled and compared to their host counterparts, with mutations in the anticodon loops highlighted in yellow. The tRNAs modeled were A) tRNA-Leu-CAA, B) tRNA-Leu-TAG, C) tRNA-Phe-GAA, and D) tRNA-Gln-TTG.

A.

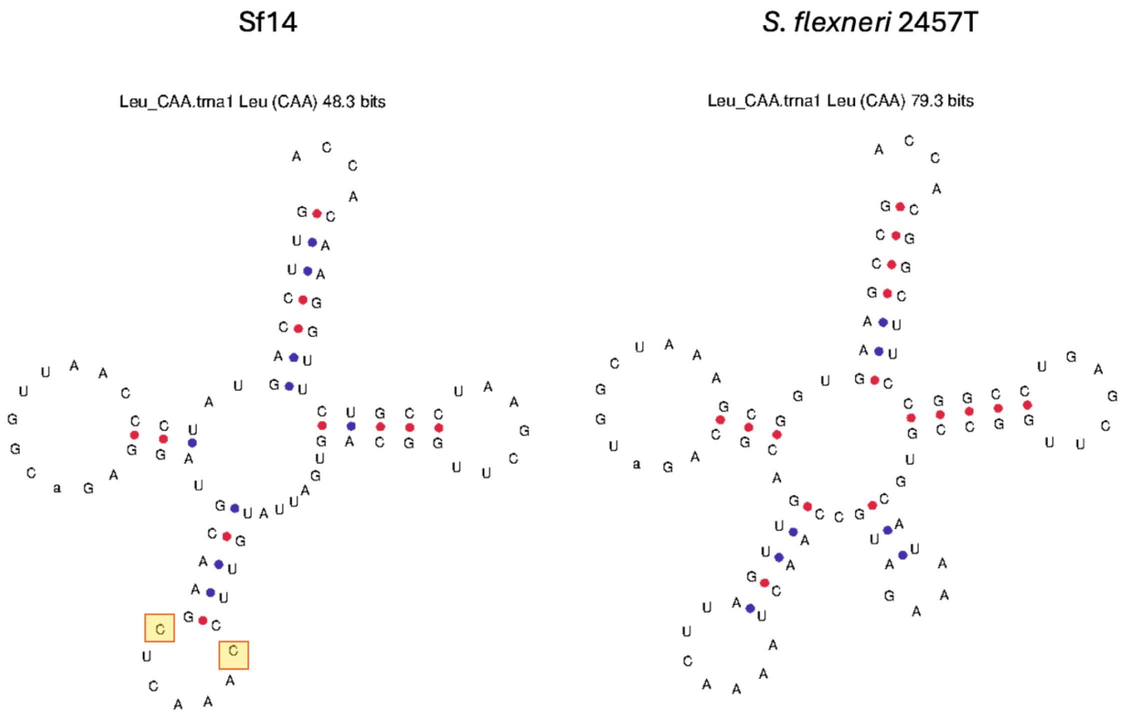

B.

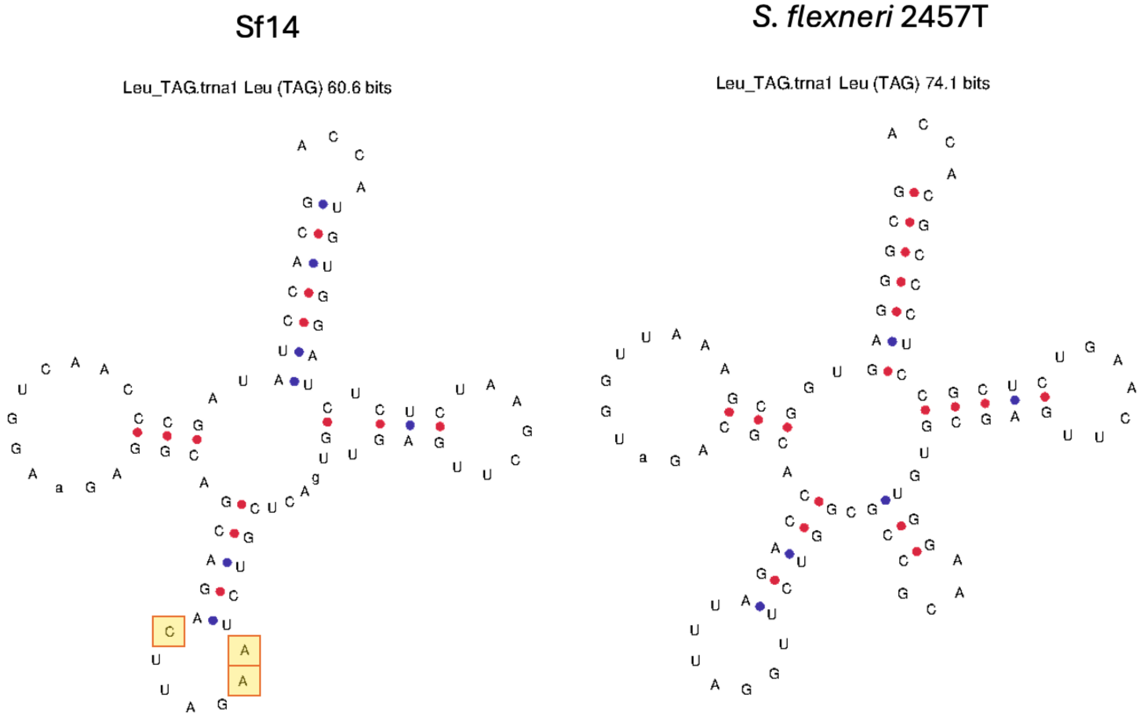

C.

Sf14

Phe_GAA.trna1 Phe (GAA) 38.8 bits

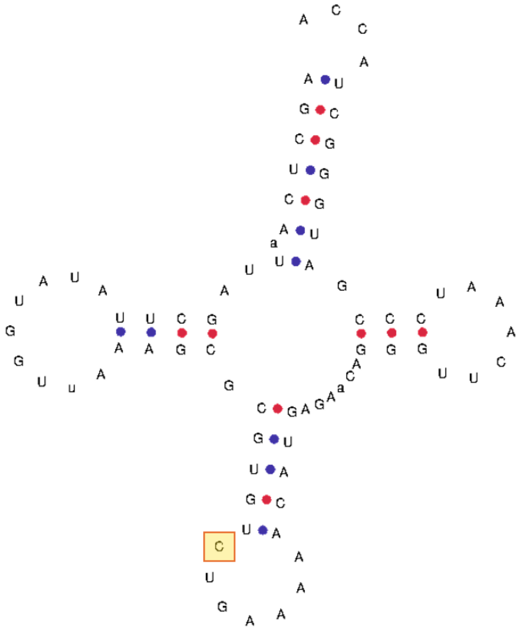

S. flexneri 2457T

Phe_GAA.trna1 Phe (GAA) 75.3 bits

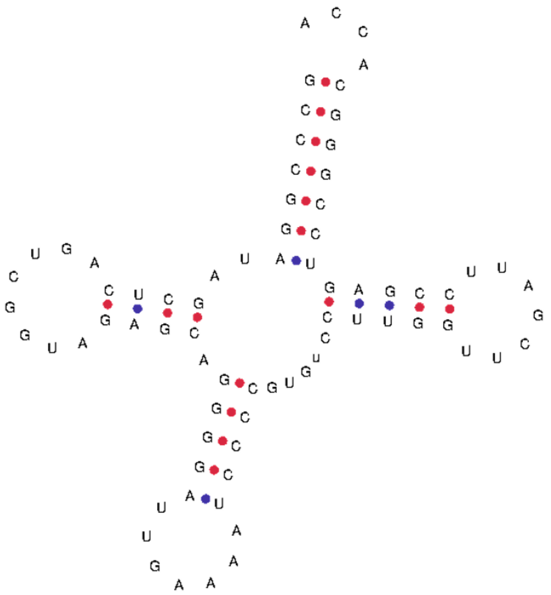

D.

Sf14

Gln_TTG_Sf14.trna1 Gln (TTG) 54.0 bits

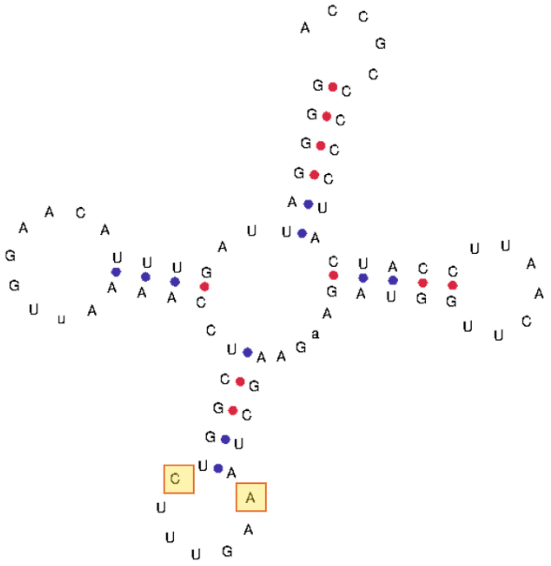

S. flexneri 2457T

Gln_TTG_2457T.trna1 Gln (TTG) 70.4 bits

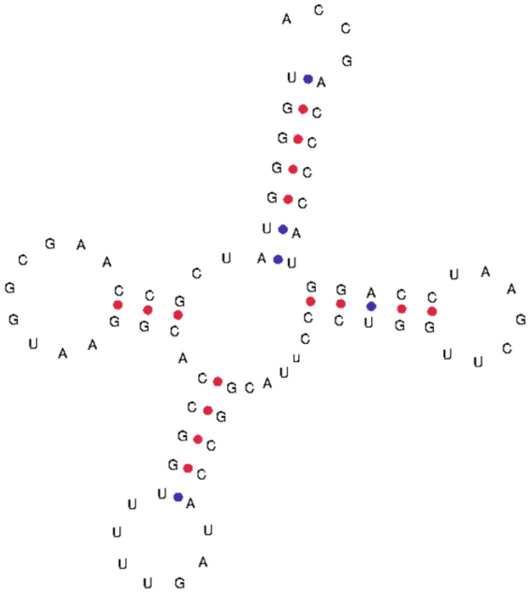

Supplement: Supplementary file 2 — Supplementary Material 2. Supplementary dataset S1: GC content analysis and statistics Values for total, GC1, GC2, and GC3 for S. flexneri 2457 T and phage Sf14 genome and individual genes. Includes tabs for all Dunn’s tests and a summary of statistics. Supplementary dataset S2: RSCU analysis and statistics Relative Synonymous Codon Usage values for the S. flexneri 2457 T and phage Sf14 genome and genes. Specific genes of interest are grouped in individual tabs. Statistics are available as raw values, then as a summary in the final tab. Supplementary dataset S3: tRNAscan-SE 2.0 output Total tRNAs predicted for S. flexneri 2457 T and phage Sf14. Includes tRNA location in the genome, predicted isotype, isotype model, anticodon, and scores. Supplementary dataset S4: tAI analysis and statistics Values for tRNA Adaptation Index for all genes in the S. flexneri 2457 T and Sf14 genome, plus genes of interest. Statistics are available in the final tab. Supplementary dataset S5: Lysine-AAG and Lysine-AAA usage analysis Genes in both S. flexneri 2457 T and the Sf14 genome with tAI ≥ 0.5 and their usage of Lysine-AAG vs. Lysine-AAA tRNAs. Supplementary dataset S6: tAI analysis using the host-only tRNA pool Values for tRNA Adaptation Index for all genes in the S. flexneri 2457 T and Sf14 genome, plus genes of interest, when using only the host-encoded tRNAs. Statistics are available in the final tab. Supplementary dataset S7: tAI analysis using the phage-only tRNA pool Values for tRNA Adaptation Index for all genes in the S. flexneri 2457 T and Sf14 genome, plus genes of interest, when using only the phage-encoded tRNAs. Statistics are available in the final tab. Supplementary dataset S8: tAI analysis using the combined tRNA pool Values for tRNA Adaptation Index for all genes in the S. flexneri 2457 T and Sf14 genome, plus genes of interest, when using both the host- and phage-encoded tRNAs. Statistics are available in the final tab. Supplementary dataset S9: comparison of anti [file 12864_2025_11998_MOESM2_ESM.pdf]
